# Extreme heat and mental health: systematic review and qualitative investigation of risk and protective factors

**DOI:** 10.1017/S0033291726105169

**Published:** 2026-07-07

**Authors:** Lea Baecker, Maisie Khan, Udita Iyengar, Alaleh Tadayon, Andrea Mechelli

**Affiliations:** Department of Psychosis Studies, Institute of Psychiatry, Psychology & Neuroscience, King’s College London, UK

**Keywords:** climate change, early intervention, extreme heat, lived experience, mental health, temperature

## Abstract

**Background:**

Extreme hot weather poses increasing risks to mental health. Yet, factors affecting vulnerability are under-researched. This mixed-method study integrates a systematic review and qualitative investigation to identify risk and protective factors for heat-related mental health issues, leading to the co-development of a screening tool. This could inform future research and, pending validation in clinical settings, support mental health professionals in assessing vulnerability among service users.

**Methods:**

We searched PubMed and Web of Science for publications on extreme heat, mental health, and risk/protective factors. In addition, we conducted six focus groups with 21 people with lived experience of heat and/or mental illness and 12 healthcare professionals. Transcripts were analyzed using thematic content analysis and informed the co-development of the screening tool.

**Results:**

Out of 764 articles identified by the systematic review, 47 were included. Evidence emerged for age, sex, existing mental illness, ethnicity, and socioeconomic status as risk factors. However, findings were inconsistent between studies, likely due to differences in study population and methodology. Protective effects included good physical health, social support, and exposure to green spaces. Our qualitative investigation identified additional risk and protective factors related to: (1) behavioral adaptability, (2) personal heat sensitivity, and (3) disparities in heat exposure. The resulting screening tool, HEAT-MH (Heat Exposure Assessment Tool for Mental Health), contains 15 questions on previous experiences of heat, general health, and lifestyle.

**Conclusions:**

The mental health impacts of extreme heat depend on a range of risk and protective factors, including demographic, socioeconomic, health, and lifestyle characteristics.

## Introduction

The frequency and intensity of heatwaves are increasing across the globe as a direct result of climate change (Thompson, Hornigold, Page, & Waite, 2018). High ambient temperatures are known to affect physical health, causing immediate risks like heat exhaustion and heat stroke, as well as longer-term issues, such as increased vulnerability to cardiovascular and respiratory diseases (Ebi et al., 2021). Recent evidence has also revealed risks to mental health. Various systematic reviews have confirmed the effect of temperature increases on mental health-related mortality, morbidity, and community well-being (Liu et al., 2021; Thompson et al., 2023; Thompson, Hornigold, Page, & Waite, 2018), for example, finding that a 1°C temperature rise was correlated with a 2.2% increase in mental health-related mortality (Liu et al., 2021). Although recent reviews have examined the impacts of extreme heat in general (e.g. Thompson et al., 2023) or in specific populations (e.g. mood disorders; Manoj, Kennedy, Liu, & Olagunju, 2025), these have primarily focused on associations between temperature and mental health outcomes, rather than factors that influence individual vulnerability. Therefore, while the mental health impact of extreme heat is now recognized, the factors that contribute to individual vulnerability remain poorly understood. A better understanding of risk and protective factors could help identify high-risk groups, guide clinical decision-making, and support early intervention during periods of extreme heat. However, the lack of screening tools specifically designed to assess heat-related mental health vulnerability currently limits clinicians’ ability to systematically identify at-risk service users.

The purpose of this mixed-method study was twofold. First, we explored risk and protective factors for heat-related mental health issues through a systematic literature review and a qualitative investigation. Here, heat-related mental health issues were defined broadly as excess mental health issues during or immediately following days of extreme hot weather, including clinical outcomes (e.g. hospital visits) and subclinical mental health outcomes (e.g. self-reported mental well-being). The qualitative investigation involved a series of focus groups with people with lived experience of extreme heat and/or mental health issues and healthcare professionals. Second, together with participants in the focus groups, we co-developed a screening tool, ‘HEAT-MH’ (Heat Exposure Assessment Tool for Mental Health), that could inform future research and, pending validation, support mental health professionals in identifying service users who are at increased risk during extreme heat.

## Methods

### Systematic review

The protocol for the systematic review followed the PRISMA guidelines (Page et al., 2021) and was registered on PROSPERO (CRD42024607805). We searched PubMed and Web of Science for publications up to August 28th, 2025, using search terms relating to heat, mental health, and risk or protective factors (full search terms in Supplementary Material S1). The inclusion criteria were: (1) studies reporting original data on one or more potential risk or protective factors; (2) published in peer-reviewed journals; (3) published in English. In this review, a risk/protective factor was defined as a baseline characteristic that makes an individual more/less likely to experience mental health issues during heat. Any kind of mental health outcome was considered, ranging from clinically diagnosed psychiatric disorders and psychiatric healthcare utilization (e.g. ICD/DSM-coded hospital admissions, emergency department visits, clinical records) to broader self-reported mental health and well-being outcomes (e.g. psychological distress, perceived well-being, emotional symptoms such as worrying). This definition was intentionally broad to allow for a wide range of study designs to be considered. In the quantitative studies, risk and protective factors were identified based on associations reported within studies, including moderation or interaction analyses, stratified subgroup analyses, and adjusted regression models; where statistical significance between groups was assessed, these findings are reported in the results. In the qualitative studies, any statements on self-reported increased or decreased vulnerability were considered. The exclusion criteria were: (1) studies reporting an association between extreme heat and mental health without reporting risk or protective factors; (2) reviews not presenting original data; and (3) studies based on temperatures averaged over a period longer than 1 day (e.g. average monthly temperature). Further information on literature screening, data extraction, and quality assessment is provided in the Supplementary Materials (S2, S3).

### Qualitative investigation

Participants for the focus groups were recruited from the general population using purposive and snowball sampling methods, e.g. via the King’s College London recruitment newsletter. While the study was open to anyone aged 18 or above, the recruitment text especially encouraged people with lived experience of mental illness and mental health professionals to participate. Further information on recruitment is provided in the Supplementary Material (S5).

**Figure 1.**
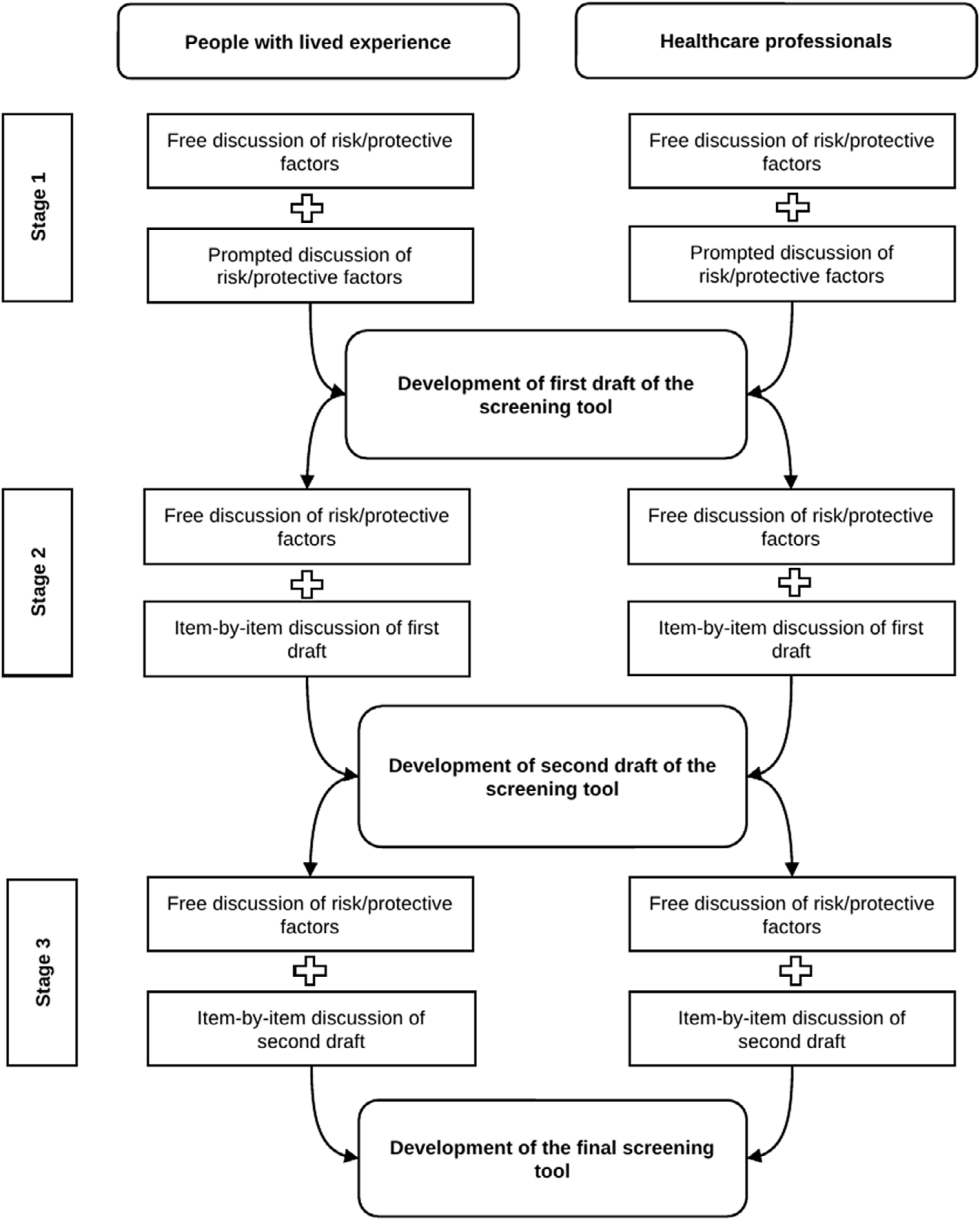
Timeline of focus groups. The six focus groups were divided into three stages. Each stage contained one focus group with people with lived experience and one with healthcare professionals. The topic guides per stage were largely the same between groups, though where people with lived experience were asked about their own experiences during extreme heat, healthcare professionals were asked about their clients. Within each stage, participants were first asked to freely name and discuss potential risk and protective factors before being asked to provide feedback on either a list of potential factors identified in a preliminary literature search (stage 1) or a draft of the screening tool developed in the previous stage (stages 2 and 3). When providing feedback on the drafts, participants were asked to rate the importance, the clarity of expression, the order of questions, and identify if any factors were missing. Further information on the topic guides is provided in Supplementary Material S4. Figure 1. long description.

Six 90 min focus groups were conducted on the King’s College London campus or virtually. They were facilitated by two researchers and audio-recorded for transcription. The groups were divided into three stages (Figure 1) with topic guides covering participants’ mental health during extreme heat (or that of their clients), and their perspectives on potential risk and protective factors (see topic guide in Supplementary Material S4). The screening tool was co-developed iteratively across the focus groups with the intended target group of existing service users of mental health services in the UK, and participants were asked for feedback on each item of the draft screening tool.

Thematic content analysis (Green & Thorogood, 2018) was used to identify factors contributing to heat-related mental health issues. Given the inverse relationship between many risk and protective factors (e.g. access to vs. lack of greenspace), a joint analytical approach was adopted. Transcripts were double-coded using NVivo software (version 14) after each stage of focus groups, and the resulting codes informed both the thematic content analysis and the co-development of the screening tool at each stage. Further information on the analytical process is provided in the Supplementary Material (S5).

The qualitative investigation received full ethical approval by the Psychiatry, Nursing and Midwifery Research Ethics Subcommittee at King’s College London (reference number LRS/DP-23/24-41409). Participants provided written informed consent.

Previous results from this qualitative investigation were presented in Baecker, Iyengar, Del Piccolo, & Mechelli (2025) (further details in Supplementary Material S4).

## Results

### Systematic review

#### Summary of systematic review

**Figure 2.**
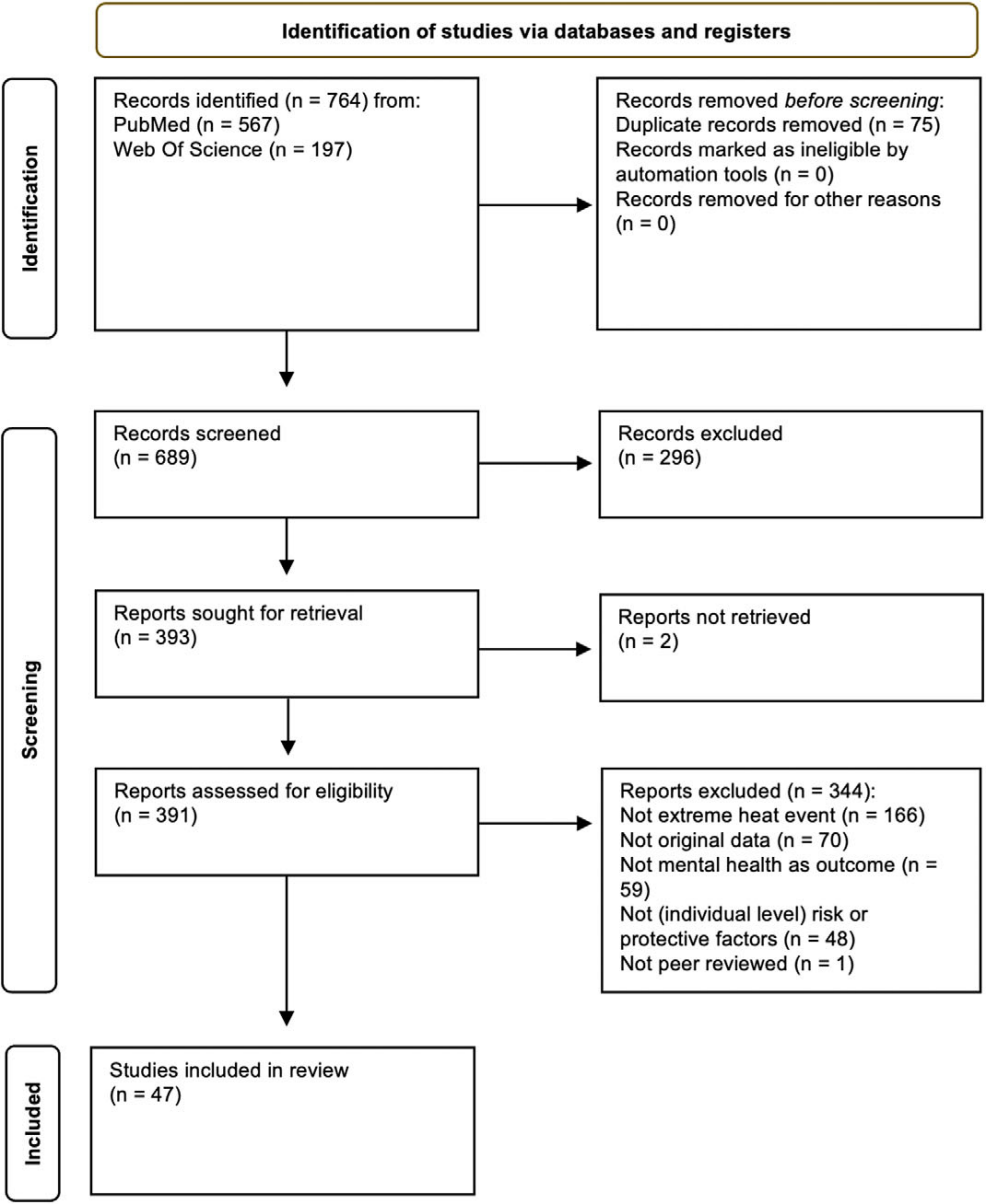
PRISMA flow diagram. Figure 2. long description.

The PRISMA flow diagram (Page et al., 2021) of the search results is presented in Figure 2 with further detail on study selection in the Supplementary Material (S6). Out of 764 search results, 47 were deemed eligible for inclusion. Forty-two studies reported on quantitative results, four studies presented qualitative findings, and one used mixed methods. Tables 1 and 2 provide an overview of the quantitative and qualitative/mixed-methods studies, respectively; further details on methods, findings, and quality ratings of individual studies can be found in the Supplementary Materials (S7, S8). The majority of quantitative studies examined clinical outcomes from administrative or clinical records (*n* = 38) or vital statistics databases (*n* = 5), such as number of hospital visits for psychiatric diagnoses or suicide data, with a minority reporting data from validated mental health scales (*n* = 6) and/or self-reported mental health impacts (*n* = 4). All qualitative studies described self-reported or perceived well-being impacts of heat. Across all included studies, the majority only examined risk factors (*n* = 40), with three studies looking solely at protective factors, and four exploring both. Findings on individual risk and protective factors are summarized below.

**Table 1.** Overview of the quantitative studies included in the systematic review Table 1. long description.

Author(s)	Study design	*N*	Population type	Mental health outcome	Location	Risk factors investigated	Protective factors investigated
Corvetto et al. (2023)	Time-series	101,452	Clinical	Hospital records	Brazil	Age, sex	N/A
Corvetto et al. (2024)	Time-series	256,406	Clinical	Hospital records	Brazil	Healthcare status, age, sex	N/A
Crank, Hondula, & Sailor (2023)	Time-series	86,672	Clinical	Hospital records	USA	Age, sex, ethnicity	N/A
Dang et al. (2022)	Time-series	7,780	Clinical	Hospital records	Vietnam	Age, sex	N/A
Dey et al. (2025)	Time-series	55,566	Clinical	Hospital records	Australia	Age, sex	N/A
Fang & Zhang (2025)	Longitudinal cohort	7,124	General	Validated scales	China	N/A	Social participation
Florido Ngu, Kelman, Chambers, & Ayeb-Karlsson (2021)	Time-series	Not specified	Clinical	Vital statistics database	60 countries globally	Age, sex	N/A
Gao et al. (2023)	Time-series	Not specified	Clinical	Hospital records	China	Age, sex	N/A
Guo et al. (2025)	Longitudinal cohort	38,087	General	Validated scales	China	Age, sex, education, residential area	Access to social, medical, and digital services
Hansen et al. (2008)	Time-series	171,614	Clinical	Hospital records	Australia	Age, sex	N/A
Hu et al. (2025)	Cross-sectional	19,852	General	Validated scales	China	Age, sex, residential area	N/A
Huebner (2022)	Cross-sectional	1,905	General	Self-reported well-being	UK, USA	Parental status, sex, age of child(ren)	N/A
Lavigne et al. (2023)	Case-crossover	9,958,759	Clinical	Hospital records	Canada	Age, sex, mental health diagnosis, air pollution, neighborhood deprivation, urbanization	Green space
Lee et al. (2018)	Time-series	166,579	Clinical	Hospital records	South Korea	Age	N/A
Li et al. (2025)	Case-crossover	12,170	Clinical	Vital statistics database	Canada	Age, sex	N/A
Liu et al. (2018)	Case-crossover	19,569	Clinical	Hospital records	China	Age, sex, residential area, occupation, marital status	N/A
Liu et al. (2022)	Time-series	5,779	Clinical	Hospital records	China	Age, sex, marital status	N/A
Mason et al. (2018)	Cross-sectional	424	General	Self-reported mental health	USA	N/A	Human, financial, physical, and social capital
Mason, Sharma, Walters, & Ekenga (2020)	Cross-sectional	426	General	Self-reported mental health	USA	Race	N/A
Min et al. (2019)	Time-series	8,438	Clinical	Hospital records	China	Age, sex	N/A
Nitschke, Tucker, & Bi (2007)	Case-series	Not specified	Clinical	Hospital records	Australia	Age	N/A
Niu et al. (2023)	Case-crossover	82,982	Clinical	Hospital records	USA	Sex, ethnicity, payment source	N/A
Nori-Sarma et al. (2022)	Case-crossover	3,496,762	Clinical	Hospital records	USA	Age, sex	N/A
Park et al. (2024)	Case-crossover	456,946	Clinical	Hospital records	South Korea	History of mental disorders, autism or intellectual disability	N/A
Park et al. (2024)	Case-crossover	14,693	Clinical	Vital statistics database	South Korea	Age, sex	Green space
Parks et al. (2023)	Case-crossover	1,512,103	Clinical	Hospital records	USA	Age, sex	N/A
Schulte, Röösli, & Ragettli (2024)	Time-series	238,596	Clinical	Hospital records	Switzerland	Age, sex	N/A
Shang et al. (2025)	Retrospective observational	5,978	Clinical	Hospital records	China	Age, sex, BMI	N/A
Shen et al. (2025)	Cohort	12,403	General	Validated scales	China	Age, sex, education, residential area	N/A
Tang et al. (2021)	Time-series	21,169	Clinical	Hospital records	China	Age, sex	N/A
Thawonmas, Kim, & Hashizume (2024)	Case-crossover	1,049,592	Clinical	Vital statistics database	Japan	Age, sex	N/A
Thawonmas, Kim, & Hashizume (2025)	Case-crossover	8,472	Clinical	Vital statistics database	Thailand	Age, sex	N/A
Ulrich, Sugg, Guignet, & Runkle (2025)	Case-crossover	324,928	Clinical	Hospital records	USA	Age, race, ethnicity, health insurance, residential area	N/A
Wang et al. (2018)	Time-series	17,744	Clinical	Hospital records	China	Age, sex, marital status	N/A
Wang et al. (2025)	Longitudinal cohort	12,316	General	Validated scales	China	Age, gender, education level, air pollution	Green/blue spaces
Li et al. (2025)	Longitudinal cohort	26,567	General	Self-reported mental health + validated scales	India	N/A	Electricity supply, water supply, ownership of cooling device
Yoo, Eum, Gao, & Chen (2021a)	Time-series	94,636	Clinical	Hospital records	USA	Age, sex, ethnicity	N/A
Yoo et al. (2021b)	Time-series	2,893,794	Clinical	Hospital records	USA	Age, sex, ethnicity	N/A
Zhang et al. (2024)	Time-series	575,505	Clinical	Hospital records	China	Age, gender, health insurance	N/A
Zhong et al. (2025)	Case-crossover	762,895	Clinical	Hospital records	China	Age, sex	N/A
Zhou et al. (2023)	Time-series	155,436	Clinical	Hospital records	China	Age, sex	N/A
Zhou et al. (2024)	Time-series	10,420	Clinical	Hospital records	China	Age, gender, marital status	N/A

*Note:* The categorization into risk or protective factors is in line with the original studies. An extended version of this table, along with detailed findings and quality ratings of each study, is provided in the Supplementary Material (S7). BMI, body mass index; N/A, not applicable.

**Table 2.** Overview of the qualitative articles included in the systematic review Table 2. long description.

Author(s)	Study design	*N*	Population type	Mental health outcome	Location	Risk factors investigated	Protective factors investigated
Goudet et al. (2024)	Qualitative (semi-structured interviews, focus groups)	80	General	Self-reported mental health	Bangladesh	Caring responsibilities	N/A
Hossain, Rana, Haque, & Al Masud (2024)	Mixed methods: Quantitative (cross-sectional surveys) + qualitative (focus groups, interviews)	Quantitative *n* = 310; Qualitative *n* ≈ 38–50	General	Self-reported mental health	Bangladesh	Quantitative: sex, education level; Qualitative: age, sex, caregiving responsibilities	N/A
Kadio et al. (2024)	Qualitative (interviews, focus groups)	71	General	Self-reported and perceived well-being	Burkina Faso	Pregnancy, postpartum period, caring responsibilities	N/A
Palinkas et al. (2022)	Qualitative (semi-structured interviews)	40	General	Self-reported and perceived mental health	USA	Caring responsibilities	N/A
Pardon et al. (2024)	Qualitative (focus groups)	31	General	Self-reported mental health	Australia	Caring responsibilities	N/A

*Note*: An extended version of this table, along with detailed findings and quality ratings of each study, is provided in the Supplementary Material (S8). N/A, not applicable.

#### Risk factors

*Age*. Thirty-six studies reported on age, with inconsistent findings. The vast majority examined clinical outcomes (*n* = 31), such as hospital attendance or suicide incidence, while a few analyzed self-reported mental health using validated scales (*n* = 4), such as depression symptoms, or qualitative interviews (*n* = 1); however, there was no discernible trend for differences in findings between these study types. In total, 11 reported a higher risk for younger to middle age groups, typically ages <60 (Corvetto et al., 2023; Florido Ngu, Kelman, Chambers, & Ayeb-Karlsson, 2021; Gao et al., 2023; Guo et al., 2025; Lavigne et al., 2023; Min et al., 2019; Shen et al., 2025; Thawonmas, Kim, & Hashizume, 2025; Wang et al., 2018; Wang, Li, & Rajagopalan, 2025; Zhou et al., 2024), whereas another 11 studies found that older adults (typically >60) were more affected (Hansen et al., 2008; Lee et al., 2018; Li et al., 2025; Liu et al., 2018, 2022; Nitschke, Tucker, & Bi, 2007; Shang et al., 2025; Tang et al., 2021; Thawonmas, Kim, & Hashizume, 2024; Zhang et al., 2024; Zhou et al., 2023). In many studies (*n* = 8), findings differed depending on the thresholds used for temperature intensity and duration (Corvetto et al., 2024; Dang et al., 2022; Hossain, Rana, Haque, & Al Masud, 2024; Niu et al., 2023; Schulte, Röösli, & Ragettli, 2024; Ulrich, Sugg, Guignet, & Runkle, 2025; Yoo et al., 2021b; Zhong et al., 2025). For example, Dang et al. (2022) found that younger ages (18–40) were most affected by temperature intensity (i.e. daily mean temperature), whereas older groups (>60) were more affected by heatwave duration. The remaining six studies reported comparable risks between age groups (Crank, Hondula, & Sailor, 2023; Dey et al., 2025; Nori-Sarma et al., 2022; Parks et al., 2023, 2024; Yoo, Eum, Gao, & Chen, 2021a).

*Sex*. Similar to age, of the 35 studies that examined sex differences, the majority used clinical outcomes such as hospital attendance or suicide incidence (*n* = 29), while a smaller number examined broader self-reported mental health and well-being outcomes from validated scales (*n* = 4) or other quantitative or qualitative approaches (*n* = 2). Overall, more studies found increased risk for men (*n* = 9) (Crank, Hondula, & Sailor, 2023; Dang et al., 2022; Gao et al., 2023; Li et al., 2025; Min et al., 2019; Nori-Sarma et al., 2022; Shang et al., 2025; Tang et al., 2021; Wang et al., 2018) than those reporting higher risk for women (*n* = 6) (Corvetto et al., 2023; Florido Ngu, Kelman, Chambers, & Ayeb-Karlsson, 2021; Guo et al., 2025; Hossain, Rana, Haque, & Al Masud, 2024; Thawonmas, Kim, & Hashizume, 2024; Zhou et al., 2023). Others observed no statistically significant difference (*n* = 13) (Dey et al., 2025; Hu et al., 2025; Lavigne et al., 2023; Liu et al., 2018, 2022; Parks et al., 2023; Schulte, Röösli, & Ragettli, 2024; Shen et al., 2025; Thawonmas, Kim, & Hashizume, 2025; Wang, Li, & Rajagopalan, 2025; Yoo, Eum, Gao, & Chen, 2021a; Yoo et al., 2021b; Zhang et al., 2024). The findings in an additional seven studies varied depending on the choice of temperature threshold, outcome measure, or region (Corvetto et al., 2024; Hansen et al., 2008; Huebner, 2022; Niu et al., 2023; Park et al., 2024; Zhong et al., 2025; Zhou et al., 2024). For instance, in a case-crossover study for heat and mental disorder-related hospital visits by Zhong et al. (2025), risks for total mental disorders were similar across sexes. However, in cause-specific analyses, women showed higher risks for anxiety and depression, while men had slightly higher risks for schizophrenia; statistical significance of group differences was not assessed.

*Pre-existing mental health conditions*. Two studies identified pre-existing mental health conditions as risk factors for heat-related clinical outcomes using hospital records. Lavigne et al. (2023) reported higher risks of mental health-related emergency department visits among individuals with mood disorders, neurotic disorders, personality behavior disorders, or developmental disorders compared to those without these conditions. Similarly, Park et al. (2024) reported that people with intellectual disabilities or a history of mental disorders had a higher risk of mental health-related emergency department admissions during heat, while people with autism did not; however, the statistical significance of group differences was not assessed.

*Ethnicity*. Six studies examined ethnicity, mainly using hospital records (*n* = 5). Findings were inconsistent. Two studies identified a higher risk for people of non-white ethnicities (Crank, Hondula, & Sailor, 2023; Niu et al., 2023), two reported no significant modification effect (Yoo, Eum, Gao, & Chen, 2021a); Yoo et al., 2021b, one had mixed results depending on the outcome measure (Ulrich, Sugg, Guignet, & Runkle, 2025). Finally, in a survey on self-reported mental health impacts, Mason, Sharma, Walters, & Ekenga (2020) found that white participants were more likely to report heat-related mental health impacts.

*Socioeconomic and environmental factors.* Socioeconomic and environmental risk factors were less frequently investigated and yielded mixed findings. One study using hospital records reported that heat had the highest impact on individuals living in materially and socially deprived neighborhoods, and on those exposed to high levels of air pollution (Lavigne et al., 2023). Five studies compared risks of residential setting using data from hospital records or obtained using validated mental health scales: three found increased vulnerability in rural areas (Guo et al., 2025; Hu et al., 2025; Ulrich, Sugg, Guignet, & Runkle, 2025), one found a higher risk in urban areas (Liu et al., 2018), and one found no difference (Shen et al., 2025). Payment source for hospital visits was analyzed in four studies as a proxy for socioeconomic status, with mixed findings (Corvetto et al., 2024; Niu et al., 2023; Ulrich, Sugg, Guignet, & Runkle, 2025; Zhang et al., 2024). For example, in the USA, commercial insurance, Medicaid, and self-pay were all found to increase the risk of mental health-related hospital visits depending on the age group of children, adolescents, or young adults, respectively (Niu et al., 2023). In Brazil, private patients had a 4.3% higher risk of emergency department visits during extreme heat (99th percentile), while public patients were at a 7.5% increased risk during moderate heat (90–99th percentile) (Corvetto et al., 2024). Education level was also examined in three studies on self-reported mental health using validated scales; here, one study suggested a greater risk for those with a lower education level (Guo et al., 2025), one for those with a higher education (Shen et al., 2025), and one reported no difference (Wang, Li, & Rajagopalan, 2025).

*Caring responsibilities.* Six studies explored caring responsibilities or parental status as a risk factor, all using self-reported mental health or well-being outcomes. In the five qualitative studies, concerns about caregiving – especially for children – briefly emerged in broader discussions of heat or climate change, e.g. in relation to women’s mental health (Goudet et al., 2024; Kadio et al., 2024; Pardon et al., 2024) or heat adaptation in low-income communities (Palinkas et al., 2022). Notably, in cross-sectional surveys in the UK and USA by Huebner (2022) on self-reported well-being impacts, parents were not more worried about their homes overheating than non-parents. Since heat was not defined and the discussion of risk factors was only brief in the qualitative studies, these studies were rated as low quality for the purposes of this review (Supplementary Material S8).

#### Protective factors

Only seven studies examined protective factors, including good health, social support, and access to green/blue spaces, on a range of clinical and self-reported mental health outcomes. For example, surveying US residents in low- and moderate-income areas on self-reported health impacts, Mason et al. (2018) identified good physical health and social cohesion as statistically significant protective factors. Similarly, among older adults in China, social participation (incl. visiting friends) and/or access to social, medical, or digital services buffered the effects of heatwaves on depressive symptoms, assessed using validated mental health scales (Fang & Zhang, 2025; Guo et al., 2025). Three studies found that exposure to green and/or blue spaces was protective (Lavigne et al., 2023; Park et al., 2024; Wang, Li, & Rajagopalan, 2025), e.g. suicide risk examined in a vital statistics database was lower in districts with more green space (Park et al., 2024).

### Qualitative investigation

#### Sample characteristics

Thirty-three participants took part across the six focus groups run in June and July 2024 (*n* = 21 people with lived experience, *n* = 12 healthcare professionals). Participants in the lived experience group had past experience of extreme heat and the majority also had a history of mental illness (68.2%). Further information on sample demographics is provided in the Supplementary Material (S9).

#### Results of thematic content analysis

We developed three overarching themes relating to (1) ability to adapt behavior during heat, (2) personal heat sensitivity, and (3) disparities in heat exposure. All information provided in this section directly arose from participant statements. A visualization of the themes and sub-themes, as well as additional quotes, is provided in the Supplementary Materials (S10, S11).

*Theme 1: Ability to adapt behavior.* Participants discussed various mental, cognitive, and physical factors that may limit a person’s ability to recognize the impact of heat and adjust their behaviors to cope. In terms of mental and cognitive factors, healthcare professionals noted that service users with a severe learning disability or mental illness might require additional support to understand the need for adaptation in hot weather. One participant explained:

*When you work with people who have psychosis, they might not be able to tell that it’s really hot outside, and then they’re wearing all their jumpers […], but then they’re getting really angry and irritated.*

A related factor limiting adaptability is a lack of knowledge about coping mechanisms, which was highlighted by several participants. Conversely, knowing how to manage heat was seen as highly protective. Many participants – drawing from experience in hotter countries – noted that those accustomed to high temperatures often cope better.

The risk from lack of knowledge may be compounded by lack of social support, leaving no one to turn to when struggling. Participants considered social isolation a major risk factor, especially during prolonged heatwaves. In contrast, a strong social support system was seen as protective by providing emotional support (e.g. sharing struggles with friends to feel less alone) and practical support (e.g. someone remembering to close the curtains before it gets hot).

The discussions highlighted that some people may recognize the need to adapt behavior but face personal or external constraints. Participants identified three key barriers: impaired mobility (e.g. due to old age or disability), inflexible work schedules (meaning one cannot avoid being outside during heat), and inability to afford cooling products (e.g. fans). Several healthcare professionals also highlighted the heat risk to inpatients, explaining that UK hospitals often lack air-conditioning or sufficient ventilation. Conversely, having control over one’s schedule was seen as protective, as some participants reflected on their own experience as students and noted that being able to reduce their workload on hot days was highly beneficial.

*Theme 2: Personal heat sensitivity.* Participants reported that some people experience negative health impacts at lower temperatures than others, either because they are more sensitive to temperature or are less resilient to external stressors. Many participants identified risk relating to altered thermoregulation in themselves or in service users, e.g. because of medication or a physical health condition. One person with lived experience described feeling the negative effects of heat at considerably lower temperatures than others around them:

*I’ve been on medication for years already, and I had no clue why I felt the way I did during periods of really hot weather. And then I came across some article […] and I was like, ‘God, I wish I knew this earlier. I wouldn’t have beaten myself up so much.*

Participants suggested that thermoregulation may also be affected by sex-specific physiological states, such as pregnancy, the postnatal period, and menopause (including perimenopause). These periods were described as particularly vulnerable times, both psychologically and physically, that may lower someone’s ability to cope with extreme heat. Thermoregulation was also perceived to be affected by substance use, with one healthcare professional highlighting that many substances raise core body heat and noting summer festivals as events that carry particular risk.

Participants felt that some individuals may have lower resilience to external stressors due to poor mental well-being or heightened sensitivity. They noted that for those already under significant stress, such as from mental illness, heat discomfort could trigger a crisis. Caring responsibilities were also seen as a risk factor, as they add the burden of managing another’s well-being alongside one’s own. This reduced resilience can be compounded during pregnancy and postnatal periods, as one healthcare professional illustrated through their own personal experience:

*I had my son four years ago and it was extreme heat, and I remember that I wasn’t sleeping, and it was so hot that I just didn’t see anybody for a long period. […] The isolation, the sleep, the anxiety, the discomfort […] will definitely predispose mothers who have just given birth.*

*Theme 3: Disparities in heat exposure.* Participants noted that certain living conditions (e.g. homelessness), work environments (e.g. around ovens, outdoors), and leisure activities (e.g. exercising outdoors) increase heat exposure and thus risks. Highlighting the link between living conditions and socioeconomic factors, one healthcare professional speculated about poorer insulation in social housing:

*If somebody lives in a tiny little housing association flat and it’s three floors up, then it’s much more difficult to find a cool space than if they live in a cottage with fat stone walls that keep your house cool.*

Participants highlighted access to cool spaces (e.g. parks, air-conditioned rooms) as highly protective. One person with lived experience noted that air-conditioning at home ‘*takes away the factor of extreme heat*,’ which was seen as particularly important for the quality of sleep. However, participants stressed that financial inequality exacerbates heat risk, as those with lower socioeconomic status often lacked both air-conditioning and nearby green spaces to cool down.

#### Screening tool

The preliminary screening tool HEAT-MH was co-developed on the basis of the thematic content analysis and the item-by-item discussion of the drafts in the focus group stages 2 and 3 (Figure 1). HEAT-MH consists of 15 questions across three domains: prior experiences of heat, general health, and daily life. The full tool is presented in Table 3 along with participant quotes on co-development, with further details provided in the Supplementary Material (S12).

**Table 3.** Proposed screening tool ‘HEAT-MH’ (Heat Exposure Assessment Tool for Mental Health) co-developed with focus group participants Table 3. long description.

Screening tool questions		Response	Quotes about the question development
*Section 1: Prior experiences*			
1	Compared to those around you, do you feel more susceptible to temperature changes in your environment or less able to respond to heat? For example, this could mean that you sweat more or feel easily overwhelmed in the heat.	Yes/No	*‘One question you could ask is ‘How do you react when it’s really hot?’ Maybe that could be a starting point to do a risk assessment.’ (Person with lived experience)*
2	On days of hot weather, do you tend to change your behavior or daily routine? For example, you might dress differently or not go outside as much.	Yes/No	*‘My patients in eating disorders would continue to exercise despite heat. […] I suppose there’s patients who will exercise and get to the point of feeling very faint or almost passing out, so in […] extreme heat, I feel like there’d still be that same pull.’ (HCP)*
*Section 2: General health*			
3	Are you older than 65 years?^a^	Yes/No	‘*There was no question on ‘how old are you?’ and I think that’s a key question as […] different age groups react differently.’ (Person with lived experience)*
4	Do you have a long-term physical health condition?	Yes/No	*‘The questions are very relevant, especially from first-hand experience of the difference of being a pre-cancer and post-cancer. […] Treatment like radiation and chemo has altered my tolerance for heat.’ (Person with lived experience)*
5	Do you have an existing mental health diagnosis?	Yes/No	*‘If there was a list of mental health diagnoses on there, I think that would be a good option. But the only problem with that is if a mental health disorder was missed out in that list or when having like a rare one, then that could cause issues, because someone might look through the list and then not see their diagnosis and think ‘okay, let me not declare that.’ (Person with lived experience)*
6	Is your mobility limited in any way? For example, if it gets hot in a room you are in, are you able to look for a cooler space by yourself?	Yes/No	*‘We have patients who need […] things to help them walk […] - like a Zimmer frame. […] When it’s really hot, it might just mean a lot more effort to get themselves out their rooms and stuff. So that could lead to social isolation, […] bring about depressive moods.’ (HCP)*
7	Are you currently taking any of the medications included on this list: Heart medication, Antidepressants, Benzodiazepines, Antipsychotics, Dopaminergics, Antihistamines, Anticholinergics, Decongestants, Stimulants, Anti-seizure medication/antiepileptics?	Yes/No	*‘I like that you’ve asked about medications as well, because there are some which you have to be especially cautious in the high heat.’ (HCP)*
8	Are you currently pregnant or suspect you might be?	Yes/No/Not applicable	*‘There are some people that obviously are unsure if they are pregnant or not. […] I think rewording the question [to include those that suspect they might be pregnant] is better than just saying ‘are you pregnant?” (Person with lived experience)*
9	Are you currently going through menopause or have you noticed any symptoms that could be linked to perimenopause? Symptoms include, but are not limited to, irregular periods, hot flashes, night sweats, or mood changes.	Yes/No/Not applicable	*‘I don’t think a lot of people, especially if English isn’t their first language, know what perimenopause is. […] So maybe then you need to explain what menopausal symptoms are, or what perimenopause is.’ (HCP)*
*Section 3: Daily life*			
10	Do you currently lack access to shelter that can protect you from weather exposure, such as sleeping outdoors, in a tent, or in a car?	Yes/No	*‘If you’re homeless, for example, that means you have little to no options to protect yourself from the effects of the weather.’ (Person with lived experience)*
11	Does your housing tend to get uncomfortably hot during hot weather without access to cooler spaces?	Yes/No	*‘It depends if you’re trying to focus on just a number that it produces for screening or whether also you’re trying to identify, as a clinician, areas which could be improved. If you’ve got no fans, that’s quite an easy thing to pinpoint for improvement. But if you’re just trying to come up with a screening tool as a risk number, then you can just combine [the list of housing options] into one question.’ (HCP)*
12	During hot weather, do you feel you have little or no support from others to help you stay cool and safe?	Yes/No	*‘Doesn’t really mean that if you live with someone that they’re going to be able to support you, like what if someone lives with four housemates that they never speak to? So, it could be relevant question, but maybe it could be rephrased - explain more […] whether people would have support, or someone else who’s thinking ‘Oh, OK. It’s going to be really hot today. First thing we do, let’s close the curtains or let’s just not even open them today when we wake up.” (HCP)*
13	Are you a carer for someone? This may include children, elderly adults, or someone living with a mental illness.	Yes/No	*‘If you’re struggling with your mental health to the extreme heat, but you’ve also got — it could be a child or any kind of dependent […] — that would be an added responsibility, an added pressure. I know that my son gets extremely irritable when it’s very hot and I’m irritable and so together it’s a lot to regulate.’ (HCP)*
14	If you work, is it difficult to keep cool at your workplace during hot weather? For example, you might work outside or around hot ovens.	Yes/No/Not applicable	*‘Whether you work outside or not, because maybe people’s work is physically straining, but it’s inside in a really well air-conditioned warehouse versus someone who might be working on a building site or something. […] Let’s say working at a market stall on one of the hottest days of the year and you’re outside in the sun for 8 hours — even if you’re not actually moving, that’s still really dangerous.’ (HCP)*
15	Do you regularly drink more than 14 units of alcohol per week during periods of hot weather?^a^ One pint of lower strength lager/beer/cider is 2 units; one pint of higher strength beer is 3 units; one standard glass of wine is 2.1 units.	Yes/No	*‘Yeah, it’s relevant. Alcohol affects the speed of sweating, affects the body temperature, which in turn can affect your thinking, your mental health, your behaviour.’ (Person with lived experience)*

*Note:* Pending validation, HEAT-MH is intended for use by mental health professionals within UK outpatient and community mental health services to help identify individuals who may be particularly vulnerable to heat-related mental health difficulties. The tool is intended to complement existing clinical judgment and support preventative interventions, care planning, and resource allocation during heat. This HEAT-MH prototype was designed for adult mental health service users with capacity to understand the questions. However, it will require validation before potential use in clinical settings and may also require additional adaptation and evaluation for use in different populations or service settings. The table lists the questions, suggested response options, and participant quotes from the co-development of the individual questions. Further supportive quotes on the content are provided in the Supplementary Material (S12). Service users are assigned a risk score on a scale of 0–15, where ‘yes’ and ‘no’/‘not applicable’ responses are counted as 1 and 0 points, respectively. Therefore, a higher score is proposed to correspond to higher risk. HCP, healthcare professional.

a Specific thresholds for age and alcohol consumption were not discussed during the focus groups. The values here were informed by the systematic review and general health guidance, respectively.

Questions were grouped into domains by the researchers and ordered by general importance based on participant feedback. The final selection, phrasing, and scoring of questions, as well as the naming of the tool, were carried out by the researchers to create a simple and time-efficient screening tool. The selection was based on the following key criteria: (1) a binary response (yes/no), (2) clear relevance to heat-related risk, and (3) appropriateness for the target population, i.e. adult service users in mental health services with the capacity to understand the questions. As a result, some risk factors that were discussed in the focus groups, such as severe learning disability or use of illicit substances, were not included.

HEAT-MH generates a risk score on the scale of 0 to 15, with each ‘yes’ response contributing one point. We propose the following risk categories: scores <5 indicate low risk, 5–10 suggest medium risk, and scores >10 indicate high risk. Future research is needed to evaluate potential redundancies amongst the questions, determine the optimal weighting for each item, and validate the proposed thresholds for the different risk categories.

The majority of participants expressed positive views on the development and potential clinical use of a screening tool for extreme heat and mental health, such as HEAT-MH. While a few noted that clinicians often know their clients well enough to assess risk informally, HEAT-MH was seen as a way to raise awareness of heat-related mental health risks and facilitate clinician-client dialogue. Some healthcare professionals emphasized the time constraints in clinical settings, underscoring the need for a brief, efficient tool.

## Discussion

This study used a mixed-method approach to explore risk and protective factors for heat-related mental health issues, leading to the proposal of the screening tool HEAT-MH for researchers and clinicians to identify the most vulnerable service users.

The systematic review revealed some evidence for age, sex, existing mental illness, ethnicity, socioeconomic status, and caring responsibilities as risk factors; however, the results were inconsistent across studies. This inconsistency may reflect the substantial methodological heterogeneity across studies, including differences in location, demographics (e.g. sex, age, socioeconomic status), definitions of heat exposure, outcomes, and analytical approaches. Notably, the review included both clinical and subclinical mental health outcomes, and the distinction between subclinical distress and clinically significant psychopathology was not always clear within studies. Furthermore, studies differed in whether they used data on healthcare utilization from administrative or clinical records or self-reported mental health data from surveys or interviews. This may have contributed to inconsistent findings on some risk/protective factors, particularly socioeconomic status. Healthcare utilization is unlikely to capture the full underlying burden of heat-related mental health impacts, as it may also be influenced by differences in healthcare access. More broadly, associations between demographic factors (e.g. socioeconomic status, ethnicity, age) and mental health outcomes may vary across populations according to contextual factors such as occupational heat exposure, housing quality, social support, and local access to cooling or shaded spaces.

As previously noted by Thompson et al. (2023), the heterogeneity of findings highlights the importance of using local data to inform policies. While previous systematic reviews identified adults over 65 as consistently vulnerable to heat-related poor mental health issues (Liu et al., 2021), our findings suggest a more nuanced relationship between age and vulnerability. Specifically, different age groups may be at risk depending on the mental health outcome or the presence of other risk factors like socioeconomic status (Corvetto et al., 2024; Niu et al., 2023). For example, younger ages appeared to be at higher risk for suicide (Florido Ngu, Kelman, Chambers, & Ayeb-Karlsson, 2021), whereas older adults were more vulnerable to depression (Zhou et al., 2023). Similarly, people of working age are more likely to be exposed to higher temperatures at work, specifically those reliant on outdoor work. The present study did not find a clear sex difference, although men seemed to be at slightly increased risk, possibly also due to greater heat exposure at work (Liu et al., 2021; Thompson, Hornigold, Page, & Waite, 2018). In contrast, while there were only two studies specifically looking at pre-existing mental illness (Lavigne et al., 2023; Park et al., 2024), they highlight this group as particularly vulnerable in line with a previous review (Thompson, Hornigold, Page, & Waite, 2018). However, many included studies on heat-related hospital utilization stratified by diagnostic category without distinguishing between pre-existing diagnoses and diagnoses identified during the heat-related hospital presentation itself (Supplementary Material S6). Overall, while there is emerging evidence for several risk factors for heat-related mental health impacts, further research using clearly defined outcome measures and more consistent methodological approaches is needed.

Our qualitative investigation identified additional risk and protective factors that were not part of the systematic review, highlighting the importance of lived experience insights. The key themes identified in the thematic content analysis related to (1) ability to adapt behavior (e.g. learning disability, impaired mobility), (2) personal heat sensitivity (e.g. medication-induced thermoregulation issues), and (3) disparities in heat exposure (e.g. homelessness, access to cool spaces). Interestingly, the factors identified across the systematic review and qualitative investigation partially overlap with those for physical and general health risks (Ebi et al., 2021). This suggests that risk for physical and mental health issues may be closely linked.

The focus groups led to the co-development of the 15-item screening tool HEAT-MH to assess individual vulnerability to heat-related mental health impacts among existing mental health service users. The tool includes questions on prior experiences of heat, general health, and lifestyle. While it is designed to generate a numerical risk score, determining the weighting of individual items and an appropriate threshold for risk levels was beyond the scope of this study and should be addressed in future research, along with its overall validity and feasibility. Our primary goal was to create a user-friendly, time-efficient prototype that could potentially be integrated into routine clinical workflows following further testing and validation in real-world mental health service settings. Some focus group participants also suggested incorporating a qualitative element, noting that the tool could serve as a conversation starter to help clinicians better understand individual challenges in hot weather and discuss possible support strategies.

Currently, research and clinical frameworks lack standardized screening tools for identifying individuals vulnerable to the mental health impacts of extreme heat. Existing tools, like the Heat Vulnerability Index (Reid et al., 2009), focus on physical health risks, considering factors like age, urban living, and chronic illnesses. While some resources, such as the Extreme Heat and Mental Illness Toolkit by the University of California, San Francisco (Cooper & Fleming, 2022) or the Heatwaves and Mental Health guide by the Royal College of Psychiatrists (2025), raise awareness of mental health risks, these do not include screening tools. In this context, HEAT-MH addresses a critical research and clinical gap by offering a practical tool to assess heat-related mental health vulnerability among existing mental health service users in the UK. It should be stressed that, in its current form, HEAT-MH must be considered preliminary. While it could be used to support research on the mental health impacts of extreme heat, it is not yet suitable for clinical use. Future longitudinal studies should examine whether HEAT-MH scores predict mental health difficulties during and following periods of extreme heat, while also evaluating potential redundancies amongst the items, optimal item weightings and risk thresholds, test-retest reliability, and the acceptability and feasibility among service users and clinicians across different clinical, demographic, and geographic settings. Following such validation, we hope HEAT-MH could help mental health professionals identify individuals at greatest risk of heat-related mental health issues early on and inform resource allocation during heatwaves. For example, clinicians could conduct check-in calls with high-risk individuals and provide them with targeted guidance on heat management. Because risk could be identified ahead of periods of heat, HEAT-MH could support earlier preventative interventions aimed at reducing mental health deterioration and the burden on mental health services during heatwaves.

Our study had several limitations. For the systematic review, the number of included studies was limited for many factors, and a few had low-quality ratings, so the findings may have limited robustness. Furthermore, the majority used ambient outdoor temperature without assessing personal exposure, and a few studies did not account for the impact of other interacting weather variables, such as sunshine hours or humidity. The vast majority of studies were conducted in the Global North (see further details in Supplementary Material S6), which may limit the generalizability of findings on risk/protective factors to the Global South and lower-resource settings. Similarly, since few studies focused on Europe, it was expected that our qualitative investigation (conducted in the UK) might highlight some factors not present in the literature.

For the qualitative investigation and co-development of HEAT-MH, the demographic characteristics of our lived experience sample may affect the generalizability of the findings. The sample size was relatively small and consisted mostly of highly educated women; as suggested in the systematic review findings, vulnerability to heat-related mental health issues may be different for men or people with lower education levels. Furthermore, most participants lived in London, UK; different risk and protective factors may therefore be relevant in other countries experiencing more frequent and intense heatwaves. However, many participants had lived in hotter climates, contributing to a diverse range of perspectives within each focus group. Additionally, although many participants reported lived experience of mental illness, the sample was not designed to be representative of the broader population of UK mental health service users. Therefore, further validation of HEAT-MH in clinical populations is required. Lastly, all health impacts were self-reported and all focus groups but one took place online, which may have also influenced the findings.

## Conclusion

Our mixed-method approach identified several risk and protective factors related to demographic, socioeconomic, health, and lifestyle factors. Our findings highlight the need for targeted interventions to provide greater support to vulnerable individuals, such as people with existing mental health conditions. Healthcare providers may need to consider these risk factors during clinical appointments, potentially adjust medication regimens, and offer additional support during heatwaves. Meanwhile, the identified protective factors may be utilized in education initiatives in community settings to help mitigate the mental health impacts of extreme heat. We hope that our qualitative findings, especially the proposed screening tool, will contribute to future research and inform efforts to safeguard mental health in an increasingly hot climate.

## Supporting information

10.1017/S0033291726105169.sm001 Baecker et al. supplementary material Baecker et al. supplementary material

## Data Availability

Data are available upon reasonable request.

## Supplementary material

The supplementary material for this article can be found at http://doi.org/10.1017/S0033291726105169.

## Long descriptions

### Figure 1. Long description

The flowchart is organized into three vertical stages, with two parallel columns for participant groups: People with lived experience on the left and Healthcare professionals on the right.

* Stage 1: Both groups engage in a Free discussion of risk/protective factors, followed by a plus symbol leading to a Prompted discussion of risk/protective factors. Arrows from both groups converge on a central box: Development of first draft of the screening tool.

* Stage 2: Both groups engage in a Free discussion of risk/protective factors, followed by a plus symbol leading to an Item-by-item discussion of first draft. Arrows from both groups converge on a central box: Development of second draft of the screening tool.

* Stage 3: Both groups engage in a Free discussion of risk/protective factors, followed by a plus symbol leading to an Item-by-item discussion of second draft. Arrows from both groups converge on the final central box: Development of the final screening tool.

Vertical labels on the far left indicate Stage 1, Stage 2, and Stage 3 corresponding to each horizontal row of activities.

Navigate back to Figure 1..

### Figure 2. Long description

The flowchart is organized into three vertical phases: Identification, Screening, and Included.

1. Identification Phase:

- Top-left box: Records identified (n = 764) from PubMed (n = 567) and Web Of Science (n = 197).

- Top-right box (connected by a horizontal arrow): Records removed before screening (n = 75) due to duplicates (n = 75), automation tools (n = 0), or other reasons (n = 0).

2. Screening Phase:

- Second central box: Records screened (n = 689).

- Second right box: Records excluded (n = 296).

- Third central box: Reports sought for retrieval (n = 393).

- Third right box: Reports not retrieved (n = 2).

- Fourth central box: Reports assessed for eligibility (n = 391).

- Fourth right box: Reports excluded (n = 344) for specific reasons: Not extreme heat event (n = 166), Not original data (n = 70), Not mental health as outcome (n = 59), Not (individual level) risk or protective factors (n = 48), and Not peer reviewed (n = 1).

3. Included Phase:

- Bottom central box: Studies included in review (n = 47).

Navigate back to Figure 2..

### Table 1. Long description

The table consists of 8 columns: Author(s), Study design, N (sample size), Population type, Mental health outcome, Location, Risk factors investigated, and Protective factors investigated.

Key entries include:

* Corvetto et al. (2023 and 2024): Time-series studies in Brazil with clinical populations (N = 101,452 and 256,406) using hospital records.

* Lavigne et al. (2023): Case-crossover study in Canada with the largest sample size (N = 9,958,759), investigating risk factors like air pollution and urbanization, and green space as a protective factor.

* Nori-Sarma et al. (2022): Case-crossover study in the U S A with N = 3,496,762 clinical subjects.

* Multiple studies from China (e.g., Fang and Zhang 2025, Guo et al. 2025, Hu et al. 2025) focus on general populations using validated scales, investigating social participation and digital services as protective factors.

* Locations span globally, including Vietnam, Australia, 60 countries globally (Florido Ngu et al. 2021), U K, South Korea, Switzerland, Thailand, and India.

* Risk factors frequently include age, sex, and residential area. Protective factors are less commonly investigated but include green/blue spaces, social capital, and access to medical or digital services.

* Sample sizes range from 424 (Mason et al. 2018) to nearly 10 million (Lavigne et al. 2023).

Navigate back to Table 1..

### Table 2. Long description

The table consists of eight columns: Author(s), Study design, N, Population type, Mental health outcome, Location, Risk factors investigated, and Protective factors investigated.

* Row 1: Goudet et al. 2024. Qualitative semi-structured interviews and focus groups. N equals 80. General population. Self-reported mental health. Bangladesh. Risk factors: Caring responsibilities. Protective factors: N / A.

* Row 2: Hossain, Rana, Haque, and Al Masud 2024. Mixed methods: Quantitative cross-sectional surveys plus qualitative focus groups and interviews. Quantitative n equals 310; Qualitative n is approximately 38 to 50. General population. Self-reported mental health. Bangladesh. Risk factors: Quantitative: sex, education level; Qualitative: age, sex, caregiving responsibilities. Protective factors: N / A.

* Row 3: Kadio et al. 2024. Qualitative interviews and focus groups. N equals 71. General population. Self-reported and perceived well-being. Burkina Faso. Risk factors: Pregnancy, postpartum period, caring responsibilities. Protective factors: N / A.

* Row 4: Palinkas et al. 2022. Qualitative semi-structured interviews. N equals 40. General population. Self-reported and perceived mental health. U S A. Risk factors: Caring responsibilities. Protective factors: N / A.

* Row 5: Pardon et al. 2024. Qualitative focus groups. N equals 31. General population. Self-reported mental health. Australia. Risk factors: Caring responsibilities. Protective factors: N / A.

Navigate back to Table 2..

### Table 3. Long description

The table is titled Table 3. Proposed screening tool H E A T minus M H (Heat Exposure Assessment Tool for Mental Health) co-developed with focus group participants. It consists of four columns: Screening tool questions (split into number and text), Response, and Quotes about the question development.

Section 1: Prior experiences

* Question 1: Asks about susceptibility to temperature changes or inability to respond to heat (e.g., sweating more). Response: Yes/No. Quote from a person with lived experience emphasizes using heat reaction as a starting point for risk assessment.

* Question 2: Asks about changes in behavior or routine during hot weather. Response: Yes/No. Quote from a healthcare professional (H C P) notes that patients with eating disorders may continue to exercise despite extreme heat.

Section 2: General health

* Question 3: Age older than 65 years. Response: Yes/No. Quote notes different age groups react differently.

* Question 4: Long-term physical health condition. Response: Yes/No. Quote mentions how cancer treatments like radiation and chemo altered heat tolerance.

* Question 5: Existing mental health diagnosis. Response: Yes/No. Quote discusses the pros and cons of listing specific diagnoses.

* Question 6: Limited mobility (e.g., ability to find a cooler space). Response: Yes/No. Quote from an H C P mentions how limited mobility can lead to social isolation and depressive moods in heat.

* Question 7: Current medications (Heart medication, Antidepressants, Benzodiazepines, Antipsychotics, Dopaminergics, Antihistamines, Anticholinergics, Decongestants, Stimulants, Anti-seizure). Response: Yes/No. Quote highlights the need for caution with specific medications.

* Question 8: Pregnancy or suspected pregnancy. Response: Yes/No/Not applicable. Quote suggests including those who suspect pregnancy.

* Question 9: Menopause or perimenopause symptoms (hot flashes, mood changes). Response: Yes/No/Not applicable. Quote suggests explaining these terms for clarity.

Section 3: Daily life

* Question 10: Lack of access to shelter (sleeping outdoors, tent, car). Response: Yes/No. Quote notes homelessness leaves no options for protection.

* Question 11: Housing getting uncomfortably hot without access to cooler spaces. Response: Yes/No. Quote discusses identifying specific areas for clinical improvement like lack of fans.

* Question 12: Lack of support from others to stay cool and safe. Response: Yes/No. Quote notes that living with others does not guarantee support.

* Question 13: Being a carer for children, elderly, or those with mental illness. Response: Yes/No. Quote mentions the added pressure of regulating a dependent’s irritability in heat.

* Question 14: Difficulty keeping cool at work (outside or near ovens). Response: Yes/No/Not applicable. Quote mentions the danger of prolonged sun exposure even if not moving.

* Question 15: Drinking more than 14 units of alcohol per week. Response: Yes/No. Quote notes alcohol affects sweating and body temperature.

A footnote indicates the tool is for mental health professionals in UK outpatient services, with a risk score scale of 0 to 15.

Navigate back to Table 3..
