# Supplementary material for: Extreme heat and mental health: systematic review and qualitative investigation of risk and protective factors
Source: Psychol Med. 2026 Jul 7;56:e219. doi: 10.1017/S0033291726105169 (PMC13370187; doi:10.1017/S0033291726105169)
Supplement: Baecker et al. supplementary material [file S0033291726105169sup001.docx]

**Supplementary Materials**

Table of Contents

[S1. Full search terms for systematic review. 2](#_Toc213843842)

[S2. Systematic review – literature screening and data extraction. 2](#_Toc213843843)

[S3. Systematic review – quality assessment of included studies. 3](#_Toc213843844)

[S4. Semi-structured topic guide used in the focus groups. 6](#_Toc213843845)

[S5. Qualitative investigation – recruitment, reflexivity, and theme development. 8](#_Toc213843846)

[S6. Additional results from the systematic review. 9](#_Toc213843847)

[S7. Further details on Table 1 which can be found in the main manuscript. 10](#_Toc213843848)

[S8. Further details on Table 2 which can be found in the main manuscript. 22](#_Toc213843849)

[S9. Demographics of focus group participants. 24](#_Toc213843850)

[S10. Visual overview of the results of the joint thematic content analysis for risk and protective factors. 25](#_Toc213843851)

[S11. Results of the joint thematic content analysis for risk and protective factors with supportive quotes from participants. 26](#_Toc213843852)

[S12. Supportive quotes from participants for each question in HEAT-MH. 28](#_Toc213843853)

[References 30](#_Toc213843854)

## S1. Full search terms for systematic review.

PubMed:

(("Extreme Heat"[Mesh] OR “Hot Temperature"[Mesh] OR "extreme heat" OR "ambient heat" OR "extreme temperature" OR heatwave OR "ambient temperature" OR "climate change") AND ("Mental Health"[Mesh] OR "mental health" or "mental illness" or "mental wellbeing" OR “psychological health”) AND ("Risk Factors"[Mesh] OR “risk factor” OR "Protective Factors"[Mesh] OR “protective factor” OR preventive OR susceptibility OR vulnerability OR resilience) NOT (review[Publication Type]))

Additional filter: English language only

Web of Science:

(ALL=("Extreme heat" OR "hot temperature" OR "ambient heat" OR "extreme temperature" OR heatwave OR "ambient temperature") AND ALL=("mental health" OR "mental illness" OR "mental wellbeing" OR “psychological health”) AND ALL=("risk factors" OR risk factor OR risk OR vulnerability OR vulnerable OR susceptibility OR "protective factors" OR protective factor OR protective OR preventive OR resilience OR resilient))

Additional filters: Document type = article; English language only

## S2. Systematic review – literature screening and data extraction.

Two authors independently conducted the literature search and screened articles based on the inclusion and exclusion criteria (LB, MK). In case of agreement, the opinion of a third author was sought (AM). Extracted data included article details (authors, publication year), participant information (sample size, population group, country), methodological details (quantitative/qualitative investigation, statistical approach, outcome measure), risk factor(s) under investigations, protective factor(s) under investigation, and results for each factor. The results of the systematic review are presented in narrative synthesis. If any of the information was not included in the publication or supplemental material, this was noted as ‘not specified’ or ‘not applicable’ in the table of extracted data.

The same authors assessed quality of included studies using adapted versions of the Newcastle-Ottawa Scale (NOS)(Wells et al., 2012) and Critical Appraisal Skills Programme (CASP) Qualitative Checklist(Critical Appraisal Skills Programme, 2024) for any quantitative and qualitative studies, respectively. Further information and the full scales and results of the quality assessment are provided in the supplemental material below (S3).

As this research field is relatively novel, we did not exclude any studies based on low quality.

## S3. Systematic review – quality assessment of included studies.

The scales were adapted to better fit the scope of the systematic review with the wide range of methodological approaches. For quantitative studies, the Newcastle-Ottawa Scale (NOS)(Wells et al., 2012) was adapted to other statistical approaches (e.g., time series analyses) following previous work.(Mahakalkar et al., 2024; Zhao et al., 2017) For qualitative studies, the same items from the NOS were applied (excl. confounding factors) with two additional items from the Critical Appraisal Skills Programme (CASP) Qualitative Checklist(Critical Appraisal Skills Programme, 2024).

For the NOS question on outcome occurrence, the NOS guidelines recommend giving one star only if the outcome (e.g., diagnosis) was independently validated. In the context of our systematic review, we assigned one star to the study if it used ICD codes based on electronic health records.

It is important to note that the quality of the studies and their findings was assessed in light of the purposes of this review rather than the original study objectives; in other words, a rating of low quality is not a judgment of the overall study, but only on the specific findings relating to risk/protective factors, which may not have been the original objective of the study.

| Author(s) | Selection (How representative was the study population?) /1 | Exposure (How was the exposure to extreme heat measured?) /1 | Validation of Outcome Occurrence (How was the mental health outcome measured?) /1 | Degree of Adjustment for Confounders /2 (only applied to quantitative studies) | Additional Questions for Qualitative Studies (Instead of confounders) /2 | | Total Score /5 | Overall Quality |
| --- | --- | --- | --- | --- | --- | --- | --- | --- |
|  | *0 = No description or not representative; 1 = Truly or somewhat representative of the average exposed individual in the community* | *0 = Exposure is based on subjective or unvalidated data (e.g., self-report); 1 = Exposure is assessed using objective, validated measures (e.g., temperature monitors)* | *0 = No description of specific mental health outcomes or not standardised or Self-report with no validation; 1 = Independent or blind assessment stated in the paper, or confirmation of the outcome by reference to secure records (x-rays, medical records, etc.) or Record linkage (e.g. identified through ICD codes on database records)* | *0 = No adjustment for confounders or not discussed; 1 = Only time-dependent meteorological variables adjusted for; 2 = Additional demographic and spatial variables considered in analysis* | *Has the relationship between researcher and participants been adequately considered? No/can't tell = 0 Yes = 1* | *Was the data quality sufficiently rigorous? E.g., was there an in-depth description of the analysis process and are sufficient data presented to support the findings? No/can't tell = 0 Yes = 1* |  | *(High 4-5, Medium 3, Low 0-2)* |
| Corvetto et al. (2023) | 1 | 1 | 1 | 2 | N/A | N/A | 5 | High |
| Corvetto et al. (2024) | 1 | 1 | 1 | 2 | N/A | N/A | 5 | High |
| Crank et al. (2023) | 1 | 1 | 1 | 2 | N/A | N/A | 5 | High |
| Dang et al. (2022) | 1 | 1 | 1 | 2 | N/A | N/A | 5 | High |
| Dey et al. (2025) | 1 | 1 | 1 | 2 | N/A | N/A | 5 | High |
| Fang & Zhang (2025) | 1 | 1 | 1* | 1 | N/A | N/A | 5 | High |
| Florido Ngu et al. (2021) | 1 | 1 | 1 | 1 | N/A | N/A | 4 | High |
| Gao et al. (2023) | 1 | 1 | 1 | 2 | N/A | N/A | 5 | High |
| Goudet et al. (2024) | 1 | 0 | 0 | N/A | 0 | 1 | 2 | Low |
| Guo et al. (2025) | 1 | 1 | 1* | 2 | N/A | N/A | 5 | High |
| Hansen et al. (2008) | 1 | 1 | 1 | 1 | N/A | N/A | 4 | High |
| Hossain et al. (2024)** | 1 | 0 | 0 | 0 | 0 (Can’t tell) | 1 | 2 | Low |
| Hu et al. (2025) | 1 | 1 | 1 | 2 | N/A | N/A | 5 | High |
| Huebner (2022) | 1 | 0 | 0 | 1 | N/A | N/A | 2 | Low |
| Kadio et al. (2024) | 1 | 0 | 0 | N/A | 0 (Can’t tell) | 1 | 2 | Low |
| Lavigne et al. (2023) | 1 | 1 | 1 | 2 | N/A | N/A | 5 | High |
| Lee et al. (2018) | 1 | 1 | 1 | 2 | N/A | N/A | 5 | High |
| Li et al. (2025) | 1 | 1 | 1 | 2 | N/A | N/A | 5 | High |
| Liu et al. (2018) | 1 | 1 | 1 | 2 | N/A | N/A | 5 | High |
| Liu et al. (2022) | 1 | 1 | 1 | 2 | N/A | N/A | 5 | High |
| Mason et al. (2018) | 1 | 0 | 0 | 1 | N/A | N/A | 2 | Low |
| Mason et al. (2020) | 1 | 0 | 0 | 1 | N/A | N/A | 2 | Low |
| Min et al. (2019) | 1 | 1 | 1 | 2 | N/A | N/A | 5 | High |
| Nitschke et al. (2007) | 1 | 1 | 1 | 1 | N/A | N/A | 4 | High |
| Niu et al. (2023) | 1 | 1 | 1 | 1 | N/A | N/A | 4 | High |
| Nori-Sarma et al. (2022) | 1 | 1 | 1 | 2 | N/A | N/A | 5 | High |
| Palinkas et al. (2022) | 0 | 0 | 0 | N/A | 0 | 1 | 1 | Low |
| Pardon et al. (2024) | 1 | 0 | 0 | N/A | 1 | 1 | 3 | Medium |
| Park, Kim, et al. (2024) | 1 | 1 | 1 | 2 | N/A | N/A | 5 | High |
| Park, Moon, et al. (2024) | 1 | 1 | 1 | 2 | N/A | N/A | 5 | High |
| Parks et al. (2023) | 1 | 1 | 1 | 2 | N/A | N/A | 5 | High |
| Schulte et al. (2024) | 1 | 1 | 1 | 2 | N/A | N/A | 5 | High |
| Shang et al. (2025) | 1 | 1 | 1 | 2 | N/A | N/A | 5 | High |
| Shen et al. (2025) | 1 | 1 | 1* | 2 | N/A | N/A | 5 | High |
| Tang et al. (2021) | 1 | 1 | 1 | 2 | N/A | N/A | 5 | High |
| Thawonmas et al. (2024) | 1 | 1 | 1 | 2 | N/A | N/A | 5 | High |
| Thawonmas et al. (2025) | 1 | 1 | 1 | 2 | N/A | N/A | 5 | High |
| Ulrich et al. (2025) | 1 | 1 | 1 | 2 | N/A | N/A | 5 | High |
| Wang et al. (2018) | 1 | 1 | 1 | 2 | N/A | N/A | 5 | High |
| Wang, Hao, et al. (2025) | 1 | 1 | 1* | 2 | N/A | N/A | 5 | High |
| Wang, Li, et al. (2025) | 1 | 1 | 1* | 2 | N/A | N/A | 5 | High |
| Yoo, Eum, Gao, et al. (2021) | 1 | 1 | 1 | 2 | N/A | N/A | 5 | High |
| Yoo, Eum, Roberts, et al. (2021) | 1 | 1 | 1 | 2 | N/A | N/A | 5 | High |
| Zhang et al. (2024) | 1 | 1 | 1 | 2 | N/A | N/A | 5 | High |
| Zhong et al. (2025) | 1 | 1 | 1 | 2 | N/A | N/A | 5 | High |
| Zhou et al. (2023) | 1 | 1 | 1 | 2 | N/A | N/A | 5 | High |
| Zhou et al. (2024) | 1 | 1 | 1 | 2 | N/A | N/A | 5 | High |

**CESD-10, PHQ-9 and GAD-7 were scored 1 despite being self-report measures, because they are validated instruments that are likely to reflect mental health in line with the aims of this systematic review.*

***Mixed-methods study therefore used all questions with a maximum rating of 7*

## S4. Semi-structured topic guide used in the focus groups.

This topic guide was developed based on a preliminary literature search and the aims of the wider research project. The topic guide had three main objectives to be reported in three distinct manuscripts: (i) to identify mechanisms underpinning the mental health impact of extreme heat (Baecker et al. (2025)), (ii) to co-develop a screening tool (present manuscript), and (iii) to co-develop a set of recommendations (Baecker & Khan et al., submitted). A separate analysis was conducted for each objective using the full transcripts.

1. [For healthcare professionals only:] How does extreme heat affect your clinical practice?
2. How does extreme heat affect you, specifically your mental health? [For healthcare professionals: How does extreme heat affect the service users under your care, specifically their mental health?]
3. What do you think are the main explanations for the effects of extreme heat on mental health? Does extreme heat influence mental health through physical changes in your body, through changes in your everyday behavior, through changes in your surrounding environment, or something else?
4. What are risk factors that make someone more likely to experience mental health issues during extreme heat?
5. What are protective factors that make someone less likely to experience mental health issues during extreme heat?
6. [Focus groups in stage 1] We will now review a list of risk factors/protective factors identified in a literature search. Based on your experience, which ones do you think are most and least relevant? Do you think any important factors are missing from this list?
   1. Housing conditions (e.g., temperature at home, lack of shelter)
   2. Work conditions (e.g., temperature at workplace, physically straining work)
   3. Pre-existing physical or mental health conditions
   4. Medications
   5. Limited or no access to temperature-controlled spaces
   6. Limited access to healthcare
   7. Social isolation
   8. Age
   9. Concerns about climate change
7. [Focus groups in stages 2/3] We will now review a list of questions for a potential screening tool that clinicians may use to identify their most vulnerable clients. These questions were developed based on previous focus groups. Which questions do you think are most and least relevant? Is their phrasing clear and appropriate? Do you think any questions are missing?
8. What are coping strategies you have or wish you could have during extreme heat? [For healthcare professionals: What are coping strategies you know service users use during extreme heat to improve/maintain their mental wellbeing? What do you recommend to them? Feel free to also discuss any coping strategies that you think are maladaptive or ineffective.]
9. [Focus groups in stage 1] We will now review a list of potential coping strategies identified in a literature search. Based on your experience, which ones do you think are most and least relevant? What are your personal strategies for this factor (e.g. what are your strategies for keeping sufficiently hydrated)? What would you recommend to someone struggling with this factor? [For healthcare professionals: Do you talk to the service users under your care about this factor? If yes, how? What would you recommend to someone struggling with this factor? Do you think any important factors are missing from this list?]
   1. Housing conditions (e.g. shading, insulation)
   2. Hydration
   3. Weather-appropriate clothing
   4. Access to temperature-controlled spaces
   5. Seeking out greenspaces, specifically shade in greenspaces
   6. Use cooling products (e.g., fans, air-conditioning, cold shower)
   7. Avoiding spaces that tend to be hot (e.g., public transport, crowded spaces)
   8. Avoiding strenuous activities
10. [Focus groups in stages 2/3] Based on our previous focus groups, we drafted a set of recommendations to improve mental wellbeing during extreme heat. Once a clinician has identified a service user to be at higher risk of experiencing mental health issues during heat, they could use these as a light-touch intervention. For each recommendation, please let us know whether you think this point is relevant/helpful and should be included in the final set of recommendations.

## S5. Qualitative investigation – recruitment, reflexivity, development of themes and screening tool questions.

Recruitment was conducted using purposive and snowball sampling methods targeting the general population. From the pool of people who completed an online expression-of-interest form, the research team purposively selected participants to ensure variation in age, gender, and ethnicity within each group. Priority was given to (a) individuals with a history of mental illness and/or mental health professionals, and (b) at the first stage, those indicating they could attend in person.

Stages 1 and 2 of the focus groups were moderated by LB (female, MSc) and UI (female, PhD) who were a research assistant and lecturer working at King’s College London. Stage 3 of the focus groups was moderated by LB and an MSc student (male, BSc) from the Early Intervention in Psychosis MSc course at King’s College London. The focus groups were supported by three additional students (female, BSc) from the Early Intervention in Psychosis MSc course who had some prior training and experience in qualitative methods and were writing their dissertations on this topic. Researchers were interested in understanding the mechanisms, risk factors, and coping strategies associated with extreme heat impacts on mental health, with a particular focus on the lived experience perspective. Participants were aware that the motivation behind the study was to produce a screening tool to identify individuals most vulnerable to extreme heat.

Themes were developed following the thematic content analysis approach described in Green and Thorogood (2018). The analysis was primarily deductive, as the aims of the analyses were predefined and some of the risk and protective factors were prompted; however, there were inductive elements, as new themes and codes arose from the open discussions with the participants. Two separate coding frames were created for lived experience and healthcare professional groups to capture and compare perspectives. This was done iteratively throughout the three stages of the focus groups in discussions between two authors (LB, AT) and three additional MSc students.

After completion of all focus groups, three authors (LB, MK, & AM) further familiarized themselves with the data through repeated reading of the transcripts. LB and MK refined the initial coding frames and identified codes related to risk and protective factors for mental health impacts of heat. Through ongoing discussions between LB and MK, relevant codes from the coding frames were systematically grouped into joint categories and then clustered into potential themes by identifying patterns, shared topics, and relationships within the data. The draft themes were reviewed against the full dataset to ensure they reflected the range and depth of participants’ views. Themes and sub-themes were then refined and finalised in consultation with a third author (AM).

Based on iterative feedback on the screening tool items throughout the three stages of the focus groups, LB and MK drafted the final set of questions and response options in consultation with AM. The proposed screening tool ‘HEAT-MH’ was intentionally developed as a broad prototype for adult users of UK mental health services rather than a specific diagnostic or service subgroup. This reflected focus group discussions highlighting that vulnerability to heat-related mental health issues may occur across diverse mental health experiences and service contexts. Based on these discussions, the prototype was limited to adult service users with capacity to understand the question phrasing, as participants in the healthcare professional groups noted that individuals with cognitive impairments may require different forms of support beyond the scope of the current tool.

## S6. Additional results from the systematic review.

Several studies were excluded from the systematic review because they explored ambient temperature without explicitly looking at heat, e.g., Bundo et al. (2021, 2023), or daily temperature range rather than minimum/mean/maximum temperature, e.g., Cohen et al.(2024). Similarly, a few studies were excluded because only conducted an analysis of risk/protective factors for general temperature increases, while the analysis of extreme heat was focused on the total sample only, e.g., Basu et al. (2018), Runkle et al. (2024), Sung et al. (2013). Further studies were excluded because they did not focus on heat events but instead averaged temperature of a longer period of time, e.g. months (Pailler & Tsaneva, 2018) or seasons (Bao et al., 2025). Additional studies were excluded because they focused on community- or population-level rather than individual-level risk, e.g., Dumont & Mathis(2023), Xu et al. (2020).

It is important to note that this manuscript defined risk/protective factors as baseline characteristics that make an individual more/less susceptible to experiencing mental health issues related to heat. The majority of included time-series studies also stratified hospital visits by diagnostic category (Corvetto et al., 2023; Dang et al., 2022; Gao et al., 2023; Hansen et al., 2008; Lee et al., 2018; Niu et al., 2023; Nori-Sarma et al., 2022; Yoo, Eum, Roberts, et al., 2021; Zhong et al., 2025). These analyses indicate which types of mental health outcomes were more sensitive to heat exposure. However, because these studies did not assess whether a diagnosis was present prior to the heat exposure, diagnosis type in this context was understood as an outcome subgroup rather than a baseline risk factor.

The most frequently studied risk factors were age and sex, though findings across all factors were mixed and appeared to be highly dependent on study population, methodology, and definition of heat. Very few studies examined risk or protective factors as a primary aim; instead, they were usually addressed in subgroup analyses (see methodological breakdown in supplementary materials S7-S8). The majority examined the association between temperature and mental health-related hospital visits, often using either a time-series approach (n=19) or a case-crossover framework (n=11). The time-series analyses generally employed distributed lag non-linear models to capture delayed heat effects, whereas case-crossover designs compared frequency of mental health-related hospital visits during extreme heat and regular temperatures within the same participants. Studies using these methodologies were rated as high quality (S3, S7). There were also five qualitative investigations, including one mixed-methods approach, which were rated as low to medium quality (S3, S8).

In terms of geographical distribution, studies were most commonly conducted in Asia (n=24) (Dang et al., 2022; Fang & Zhang, 2025; Gao et al., 2023; Goudet et al., 2024; Guo et al., 2025; Hossain et al., 2024; Hu et al., 2025; Lee et al., 2018; J. Liu et al., 2022; X. Liu et al., 2018; Min et al., 2019; Park, Kim, et al., 2024; Park, Moon, et al., 2024; Shang et al., 2025; Shen et al., 2025; Tang et al., 2021; Thawonmas et al., 2024, 2025; S. Wang et al., 2018, 2025; Zhang et al., 2024; Zhong et al., 2025; Q. Zhou et al., 2024; Y. Zhou et al., 2023), followed by North America (n=13) (Crank et al., 2023; Huebner, 2022; Lavigne et al., 2023; Li et al., 2025; Mason et al., 2018, 2020; Niu et al., 2023; Nori-Sarma et al., 2022; Palinkas et al., 2022; Parks et al., 2023; Ulrich et al., 2025; Yoo, Eum, Gao, et al., 2021; Yoo, Eum, Roberts, et al., 2021), Australia (n=4) (Dey et al., 2025; Hansen et al., 2008; Nitschke et al., 2007; Pardon et al., 2024), South America (n=2) (Corvetto et al., 2023, 2024), and Africa (n=1) (Kadio et al., 2024). Only two studies included a European sample (Huebner, 2022; Schulte et al., 2024), and one study conducted a global comparison (Florido Ngu et al., 2021).

## S7. Further details on Table 1 which can be found in the main manuscript.

Overview of the quantitative studies included in the systematic review. A summary table was provided in the main manuscript (Table 1). The categorisation into risk or protective factors is in line with the original studies. If a study reported on different types of climate events or climate change in general, only findings related to heat events were extracted. The table includes an overall quality score for each study; an item-by-item breakdown of this score is provided in supplementary materials above (S3).

*Abbreviations: CESD, Center of Epidemiologic Studies Depression Scale; DEHH, daily excess hourly heat; ICD, International Classification of Diseases; ICE, Index of Concentration at the Extremes; MDP, mental disorders of pregnancy; N/A, not applicable; NDVI, Normalized Difference Vegetation Index; OR, odds ratio; PHQ, Patient Health Questionnaire; PMAD, perinatal mood and anxiety disorders; RR, relative risk; SMI, severe mental illness*

| Study | Study design and analysis | N | Population type | Location and study period | Definition of heat | Mental health outcome | Risk factors investigated | Protective factors investigated | Findings | Quality rating (scale 1-5) |
| --- | --- | --- | --- | --- | --- | --- | --- | --- | --- | --- |
| Corvetto et al. (2023) | Time-series analysis using quasi-Poisson regression and a distributed lag non-linear model, stratified by subgroups | 101,452 | Clinical: Emergency department visits for mental health and suicide attempts | 9 public emergency healthcare units in Curitiba, Brazil (2017-2021) | 99th percentile (24·5°C) of the daily mean temperature | Mental health-related emergency department visits or suicide attempt data obtained via digitised anonymous health records | Age (0-17, 18-64, ≥65); sex (male, female) | N/A | There was a higher cumulative risk of emergency department visits during extreme heat for ages 18-64 (RR 1·16 at lag0-10), whereas those ≥65 had reduced risk (RR 0·77 at lag0-4). Women were at higher risk than men (peaked at lag0-6 with a RR of 1·20 and persisted until lag0-9). Statistical significance of subgroup differences was not assessed. | 5 (high) |
| Corvetto et al. (2024) | Time-series analysis using quasi-Poisson regression and a distributed lag non-linear model. Meta-regression subgroup analysis was used to compare public vs private status, and these were further stratified by age and sex. | 101,452 (public) vs 154,954 (private) | Clinical: Emergency department visits for mental health and suicide attempts | 9 public emergency healthcare units and 1 private hospital in Curitiba, Brazil (2017-2021) | Extreme heat: 99th percentile (24·5°C) of the daily mean temperature; Moderate heat: 90-99th percentile | Mental health-related emergency department visits or suicide attempt data obtained via digitised anonymous health records. Diagnosis of mental health ICD-10 category was recorded on admission. | Public vs private healthcare (as proxy for socioeconomic status). Healthcare status further stratified by sex (private female vs public female; private male vs public male), and age (public vs private 18-64, public vs private ≥65) | N/A | Extreme heat: Private patients had a 4·3% higher risk for mental health-related emergency department visits than public patients; For women, private patients had 2·9% increased risk compared to public patients. For men, private patients had 6·7% increased risk vs public patients. For age group 18-64, private patients had 3·6% increased risk vs public patients. For age group ≥65, private patients had 33·4% increased risk vs public patients.  Moderate heat: Public patients at 7·5% higher risk than private patients. For women, public patients had 13·7% increased risk compared to private patients. No differences were found for public vs private male patients. For age group 18-64, public patients had 10·0% increased risk vs private patients. For age group ≥65, private patients had 3·9% increased risk vs public patients. Statistical significance of subgroup differences was not assessed. | 5 (high) |
| Crank et al. (2023) | Time-series analysis using a distributed lag non-linear model, stratified by subgroups | 86,672 | Clinical: Schizophrenia hospitalisations | Maricopa County, Arizona, USA (2006-2014) | Daily min. temperature of 30°C (i.e., 82nd percentile minimum temperature in the study region during June-August) | Hospitalisations for schizophrenia extracted from Department of Public Health records | Sex (male, female); ethnicity (white, non-white); age (0-17, 18-64, >64) | N/A | There was a higher risk for men particularly at lag0 and lag1. Non-white ethnicities were at higher risk, particularly at lag1. Statistical significance of subgroup differences for sex and ethnicity was not assessed.  No statistically significant age difference was found. | 5 (high) |
| Dang et al. (2022) | Time-series analysis using quasi-Poisson regression and a distributed lag linear model, stratified by subgroups | 7,780 | Clinical: Mental and behavioural disorder hospitalisations | Ho Chi Minh City, Vietnam (2017-2019) | Heatwave defined as min. 97th percentile of daily mean temperature (30·9°C) for min. 2 days | Mental and behavioural disorder hospitalisations obtained from hospital admission records | Sex (male, female); age (0-17, 18-40, 41-60, >61) | N/A | The main effect of heatwaves (temperature intensity) was strongest for men (RR 2.08). Younger ages were most affected by temperature intensity, most in 18-40 (RR 1·91), followed by 41-60 (RR 1·50); older groups (>60) were more affected by the added effect, i.e., heatwave duration (RR 1·67). Statistical significance of subgroup differences was not assessed. | 5 (high) |
| Dey et al. (2025) | Time-series analysis (generalised additive model with negative binomial distribution), stratified by subgroups | 55,566 suicidality presentations | Clinical: emergency department visits for suicidality in youth (ages 12-24) | New South Wales, Australia (November-March 2012-2019) | Heatwave: ≥3 days ≥95th percentile of daily mean temperature | Suicidality (ICD-10 codes: self-harm, suicidal ideation, drug overdose or poisoning; or free text) | Sex (male, female); age (12-17, 18-24) | N/A | No significant effect of heatwaves above daily mean temperature, with no differences by sex or age. Statistical significance of subgroup differences was not assessed. | 5 (high) |
| Fang & Zhang (2025) | Longitudinal cohort study. Multilevel fixed effect model to explore temporal association between heatwaves and depression, with social participation types as moderators. | 7124 | General: Older adults aged ≥55 years (from China Health and Retirement Longitudinal Study) | China (2015-2017) | Heatwave: ≥3 days ≥35°C daily maximum temperature | Depressive symptoms measured using CESD-10 (self-reported) | N/A | Self-reported social participation, incl. (1) volunteer participation (e.g., providing help to people, charity work) and (2) leisure participation (visiting friends, playing group games/sports, community clubs etc.) | Leisure participation mitigated the effect of heatwaves on depressive symptoms (b =̶ 0.52, p < 0.05), while volunteer participation did not. | 4 (high) |
| Florido Ngu et al. (2021) | Time-series analysis using negative binomial Poisson regression with stratified analysis for age and sex. | Not specified | Clinical: Any occurrence of suicide | 60 countries globally (1979-2016) | Heatwave defined as min. 99th percentile of local daily mean temperature for min. 4 days. | Suicide/fatal intentional self-harm obtained from World Health Organisation's mortality database | Sex (male, female); age (5-14, 15-24, 25-34, 35-54, 55-74, 75+) | N/A | Women tended to be more frequently affected by changes in heatwave counts. Younger age groups (5-14, 15-24 years) also tended to show stronger associations than older groups. Statistical significance of subgroup differences was not assessed. | 5 (high) |
| Gao et al. (2023) | Time-series analysis using a quasi-Poisson generalised additive model and a distributed lag non-linear model; stratified analyses by risk factor. | Not specified | Clinical: Mental and behavioural disorder hospitalisations | Heibei Medical University, Shijiazhuang, China (2014-2019) | 90th percentile (29°C) (used 15.3° as reference temperature, so RRs refer to the increased hospitalisation risk after 29°C compared to 15·3°.) | Mental and behavioural disorder hospitalisations obtained from mental health centre records. | Sex (male, female); age (<45, ≥45) | N/A | For sex, higher hospitalisation rate was only observed in men, not women (with cumulative effect significant to lag6). For age, higher hospitalisation rate was only observed in the younger group (<45 years) (cumulative effect significant to lag6). Statistical significance of subgroup differences was not assessed. | 5 (high) |
| Guo et al. (2025) | Longitudinal cohort study. Two-way fixed effect regression models with service use as moderators, stratified by subgroups. | 38,087 observations across 5 survey waves | General: Older adults aged ≥60 years (from China Health and Retirement Longitudinal Study) | China (2011-2020) | High temperature days were when the daily maximum heat index exceeded 90th percentile considered the combined effects of air temperature and humidity on percieved temperature | Depressive symptoms measured using CESD-10 (self-reported) | Sex (male, female); age (<75, ≥75); education level (primary and below, middle school and above), residential area (urban, rural) | Access to services in past month: participation in social services; receipt of medical services; digital service (internet) usage | Access to social, medical, and digital services can mitigate the negative mental health impact of extreme heat. Subgroup analyses found statistically significant increases in depressive symptoms for participants that were female, 60-74 years old, had lower education level, and/or were rural-living. | 5 (high) |
| Hansen et al. (2008) | Time-series analysis using Poisson regression; subgroup analyses for different diagnoses, stratified by subgroups. | 171,614 admissions (4,629 during heatwaves) | Clinical: Hospital admissions and mortalities for mental, behavioural, and cognitive disorders | Adelaide, South Australia (1993-2006) | Heatwave: ≥3 days ≥35°C daily maximum temperature | Hospital admissions and mortalities for mental, behavioural, and cognitive disorders (ICD-10) | Sex (male, female); age (15-64, 65-74, ≥75) | N/A | Subgroup analyses showed statistically significant increases in hospital admissions for mental and behavioural disorders during heatwaves among adults ≥75 years, and among men aged 15-64 years. Furthermore, subgroup analyses showed statistically significant increases in mortality for mental and behavioural disorders among adults aged 65-74 years. Other age-sex analyses showed elevated point estimates with overlapping confidence intervals. No statistical tests were conducted to compare age or sex groups directly. | 4 (high) |
| Hu et al. (2025) | Cross-sectional survey; logistic regression analyses, subgroup analyses | 19,852 | General: Adolescents aged 10-18 (from the Chinese adolescent health survey) | China (April-December 2021) | Heatwave: ≥3 days with excess heat factor (denoting short- and long-term temperature anomalies) above 0 | Depression and anxiety measured using the PHQ-9 and GAD-7 | Sex (male, female); region of school (urban, rural); school grade (junior, senior high school) | N/A | Rural students were significantly more likely to experience depression and anxiety than urban students. Sex and grade were non-significant for depression, but for anxiety alone, males were significantly more affected than females. | 5 (high) |
| Huebner (2022) | Cross-sectional online surveys; subgroup analyses using t-tests and/or regression, with equivalence testing applied to null results. | n=1905 total (UK: n=463 parents, n=492 non-parents; USA: n=449 parents, n=501 non-parents) | General: parents and non-parents | UK, USA (September 2021) | Not specified (asked about overheating on a normal summer day and during a heatwave) | Worry about home overheating | Parental status (parents, non-parents); sex (male, female); age of child(ren) (0-2, 3-5, 6-10, 11-17) | N/A | In the UK, significance testing found no differences in overheating worry by parental status, sex, or child age. In the USA, parents reported lower worry than non-parents, possibly due to greater use of air-conditioning. Furthermore, in the USA, men without children worried more than mothers, and parents of 6-10-year-olds and non-parents reported higher worry than parents of 0-2-year-olds. | 2 (low) |
| Lavigne et al. (2023) | Case-crossover study with 3-4 control periods for each participant, matched to the same day of the week and month of the admission. Conditional logistic regressions for each region were used (and multivariate meta-regression models to pool estimates) and combined with distributed lag non-linear models. Stratified analyses within models to calculate OR for risk factors. | 9,958,759 | Clinical: Mental and behavioural disorder emergency department visits | Alberta and Ontario, Canada (2004-2020) | 97·5th percentile of daily average temperature | Mental and behavioural disorder emergency department visits obtained from the National Ambulatory Care Reporting System. | Age (<18, 18–29, 30–49, ≥50); Sex (male, female); Pre-existing mental health condition (schizophrenia, mood disorder, neurotic disorder, personality behaviour disorder, developmental disorder, and dementia); Ambient air pollution (NO_2_, O_3_, PM_2.5_); Neighbourhood deprivation; Urbanisation. | Residential green space exposure (tree canopy coverage and NDVI) | For age, risk was highest for those aged 30-49 (OR 1·183) and lowest for <18s (OR 0.966). No significant effect of sex was found. For pre-existing mental health conditions, prior diagnosis of mood disorders, neurotic disorders, personality behaviour disorders, and developmental disorders were associated with significantly higher risk. Exposure to the air pollutants NO_2_ (OR 1·182) and O_3_ (OR 1·178) were associated with greater risk. For neighbourhood deprivation, highest risk was reported for those living in materially and socially deprived neighbourhoods (OR 1·181) compared to privileged neighbourhoods (OR 1·042). Effects of urbanisation were not clear.  For protective factors, tree canopy coverage attenuated impact of heat but effects of NDVI were not clear. | 5 (high) |
| Lee et al. (2018) | Time-series analysis using a quasi-Poisson regression and distributed lag non-linear model; multivariate meta-analysis to pool results across cities. Subgroup analyses were conducted to identify risk factors. | 166,579 | Clinical: emergency hospital admissions for mental disorders | Six major cities in South Korea (2003-2013) | 99th percentile (29·4°C) of daily maximum temperature | Mental health-related emergency admissions obtained from the Korean National Health Insurance Corporation. | Age (<65, >65) | N/A | Higher cumulative risk for the older group (age >65 years) with RR 1·22 over >4 lag days (compared to RR 1·138 for <65). No significance testing was reported. | 5 (high) |
| Li et al. (2025) | Time-stratified case-crossover study; conditional logistic regression, stratified by subgroups | 9,599 suicide attempts, 2,571 suicide deaths | Clinical: suicide attempts and suicide deaths | British Columbia, Canada (21st June-21st September 2004-2023; attempts only from 2012) | Hot day defined as ≥95th percentile of temperatures over previous 4 weeks (for each local health area) | Suicide attempts and suicide deaths | Sex (male, female); age (<25, 25-55, >55) | N/A | Stronger associations between hot days and attempted suicides were reported for those aged >55 (OR 1.46) and men (OR 1.26). Men also had a stronger association completed suicides by self-poison (OR 1.50). Similar associations were reported when using 2-day instead of 1-day temperature averages. Statistical significance of group differences was not assessed. | 5 (high) |
| Liu et al. (2018) | Case-crossover study with multifactor logistic regression analysis to identify risk factors for hospital visits during heatwaves. 4-6 control days per exposure day were selected to match the day of the week of the admission. | 19,569 | Clinical: Hospital visits related to mental illness | Shandong Province, Jinan, China (2010) | Heatwave: daily maximum temperatures ≥35°C for min. 3 days. | Daily hospital visits for mental illness obtained from electronic records from the mental health centre. | Sex (male, female); Age (≤64, ≥65); Home address (urban, rural/suburban); Occupation (outdoor worker, indoor worker); Marital status (married, other/single) | N/A | There was no significant sex difference. Older people were at higher risk (OR 3·034), as were those living in urban areas (OR 1·523), outdoor workers (OR 1·714), and single people (RR 1·709). | 4 (high) |
| Liu et al. (2022) | Time-series Poisson regression model with distributed lag non-linear model, stratified by subgroups | 5779 | Clinical: individuals with schizophrenia on case information database of Jining Psychiatric Hospital | Jining, China (2014-2020) | ≥2 days above 95th percentile of daily mean temperature | Schizophrenia hospitalisation (diagnosis of schizophrenia according to ICD-10) | Sex (male, female); age (<40, ≥40); marital status (married, other/single) | N/A | Participants aged ≥40 and those who are married appeared at slightly elevated risks, whereas no gender differences were found. However, no significance testing for subgroup analysis for heatwave-only event was reported. | 5 (high) |
| Mason et al. (2018) | Cross-sectional survey mailed to random residences. Responses were analysed using descriptive statistics and logistic regression, with qualitative responses coded and categorised into impact type. | 424 (individuals, not events) | General: residents in low- and moderate-income areas. | Knoxville, Tennessee, USA (August-November 2016) | Not specified | Self-reported perceived impact of heat on mental health by answering the question “To what extent is your mental health negatively affected by very hot temperatures in the summer?”. Responses were 1=not at all, 2=slightly or somewhat, 3=very much. | N/A | Human capital (education level and general health); Financial capital (income level and emergency savings (yes/no)); Physical capital (homeownership (yes/no) and central cooling (yes/no); Social capital (social cohesion score). | Having good/very good (self-reported) general health was negatively associated with mental health impacts of summer heat (OR 0·379, *p*=0.006). Social cohesion was also negatively associated with mental health impacts of summer heat (OR 0·710, *p*=0.004). All other factors were non-significant. | 1 (Low) |
| Mason et al. (2020) | Cross-sectional survey, chi-square test for subgroups | 426 | General: residents in low-and-moderate income areas | Knoxville, Tennessee, USA (August-November 2016) | Not specified (asked about very hot temperatures in the summer) | Self-reported impact on mental health | Race (White, Black, other) | N/A | White participants were significantly more likely to report mental health impacts of extreme heat. | 2 (low) |
| Min et al. (2019) | Time-series analysis using quasi-Poisson generalised linear model and distributed lag non-linear model; stratified analyses by sex and age. | 8,438 | Clinical: emergency hospital admissions for mental and behavioural disorders | Yancheng, China (2014-2017) | 90th percentile of apparent temperature (30·6°) | Mental health emergency admissions obtained from hospital medical records. | Sex (male, female); Age (<45, 45-60, ≥60) | N/A | There was an increased lag-specific risk for men but not women, and an increased risk for ages <45 but not 45+. Statistical significance of group differences was not assessed. | 5 (high) |
| Nitschke et al. (2007) | Case-series study; Poisson regression models | Not specified | Clinical: mental health hospital admissions and mortality | Adelaide, South Australia (1993-2006) | ≥35°C for ≥3 consecutive days | Mental health-related hospital admissions or mental-health related mortality | Age (0-4, 5-14, 15-64, 65-74, ≥75) | N/A | Mental health hospital admissions increased across all ages but ≥75 was the only statistically significant age group on its own (IRR 1.17 [1.07,1.28]) and statistical significance of age group differences was not assessed. Associations of heat and mental health-related mortality were not significant. | 4 (high) |
| Niu et al. (2023) | Case-crossover study with conditional logistic regression and distributed lag non-linear model. 3 or 4 controls were used per case, and were matched on day of the week, month, and year. Subgroup analyses separately for each age group were used to identify risk factors. | 82,982 | Clinical: mental health-related emergency department or hospital visits in children (6-11), adolescents (12-17) and young adults (18-25). | New York City, USA (June-August 2005-2011) | 95th percentile (76°F, approx. 24·4°C) compared to min. risk temperature (temperature of lowest cases). | Mental health-related emergency department and hospital encounters (with presence of primary mental health diagnosis or suicide/self-inflicted injury category) extracted from the New York Statewide Planning and Research Cooperative System. | Sex (male, female); Ethnicity (Hispanic, non-Hispanic Black, non-Hispanic White, non-Hispanic other); Payment source (commercial, Medicaid, self-pay, other) | N/A | Children: No sex difference; Higher risk for non-Hispanic Black (cumulative OR 1·43) and non-Hispanic other (OR 1·60); Higher risk for Medicaid (OR 1·48). Adolescents: No difference for sex; Higher risk for non-Hispanic Black (OR 1·21) and non-Hispanic other (OR 1·26); higher risk for commercial insurance (OR 1·25)~~.~~ Young adults: Higher risk for male (OR 1·07); Higher risk for non-Hispanic other (OR 1·12); Higher risk for Medicaid (OR 1·20) and self-pay (OR 1·33). However, heterogeneity testing found that differences between subgroups were not statistically significant. | 4 (high) |
| Nori-Sarma et al. (2022) | Time-stratified case-crossover study; conditional logistic regression with distributed lag non-linear models, stratified by subgroups | 3,496,762 visits (2,243,395 unique individuals) | Clinical: mental health emergency department visits of adults | USA (2010-2019) | 95th percentile of the county-specific temperature distribution | Emergency department visits for mental health (diagnoses according to ICD-10) | Age (18-26, 27-44, 45-64, ≥65); sex (male, female) | N/A | Associations between heat and emergency department visits were higher for men (IRR 1.10 [1.08-1.12]) than women (IRR 1.06 [1.05-1.08]). No heterogeneity across age groups was reported. Statistical significance of group differences was not assessed. | 5 (high) |
| Park, Kim, et al. (2024) | Time-stratified case-crossover study; conditional logistic regression with distributed lag non-linear model, stratified by subgroups | 456,946 | Clinical: individuals with intellectual disability, autism, or mental disorder | South Korea (June-September 2006-2021) | 99th percentile (vs 75th percentile) | Emergency department admission for mental disorders (based on ICD-10) | History of mental disorders (dementia, schizophrenia, mood, anxiety, stress) or diagnosis of autism or intellectual disability | N/A | People with intellectual disabilities (OR 1.24 [1.07-1.45]) and existing mental disorders (OR 1.18 [1.09-1.28]) had a higher risk of being admitted for mental disorders during heat, while people with autism did not (OR 0.96 [0.55-1.65]). Statistical significance of group differences was not assessed. | 5 (high) |
| Park, Moon, et al. (2024) | Space-time-stratified case-crossover study with distributed lag nonlinear functions, stratified by subgroups | 14,693 | Clinical: suicide mortality data | Seoul, South Korea (June-September 2000-2020) | Hot night indices (hot night duration and hot night excess) | Suicide | Age (<30/30-64/≥65), sex (male, female) | Green space (NDVI) | The association between hot night duration and suicide was stronger in men (RR 1.06 [1.01-1.11]) than women (RR 1.02 [0.95-1.09]), but this was not statistically significant. No significant differences were observed across age groups. Furthermore, there was a higher risk of suicide associated with night-time heat exposure in low NDVI districts than high NDVI districts. No significant differences were reported for age or sex. | 5 (high) |
| Parks et al. (2023) | Time-stratified case-crossover study; distributed lag non-linear model; conditional logistic regression; stratified by subgroups | 717,798 total hospital visits for alcohol-related disorders and 794,305 for substance-related disorders | Clinical: alcohol- and substance-related disorder cases | New York State, USA (1995-2014) | 90th percentile, 99th percentile (relative to minimum temperature) | Hospital admission for alcohol- or substance-related (cannabis, cocaine, opioids, sedatives) disorder (ICD-9-CM) | Age (0-24; 25-44; 45-64; 65+), sex (male, female) | N/A | No difference by age or sex were reported at either percentile threshold. Statistical significance of group differences was not assessed. | 5 (high) |
| Schulte et al. (2024) | Time-series analysis using distributed lag non-linear model; conditional Poisson regression, stratified by subgroups | 238,596 admissions | Clinical: emergency department admissions for mental and behavioural disorders | Switzerland (May-September 1998-2019) | 99th percentile of the daily maximum temperature (34•C) relative to disease-specific optimum temperature | Hospital admission for mental and behavioural disorders (ICD-10) | Age (0-14; 15-64; 65-74; 75-84; ≥85), sex (male, female) | N/A | The risk was higher for age groups 15-64 years and 75-84 years, while it was not significant for the other age groups. The risk was significant for both men and women, with slightly higher risk for men. Statistical significance of group differences was not assessed. | 5 (high) |
| Shang et al. (2025) | Retrospective observational study using mixed-effect logistic regression and structural equation modelling, stratified by subgroups | 5,978 patients (7,118 hospitalisations) | Clinical: Inpatients with depression | Anhui, China (2020-2023) | Heatwave: ≥3 days ≥95th percentile of daily mean temperature (other durations also analysed) | Diagnosis of depression (ICD-10) categorised into mild, moderate or severe depression based on discharge diagnosis after a hospitalisation | Sex (male, female); age (≤18 years, minors; 19–64 years, young and middle-aged individuals; ≥65 years, elderly individuals), BMI (<18 kg/m2, underweight; 18–24 kg/m2, normal weight; > 24 kg/m2, overweight). | N/A | For a 3d heatwave, men (3d: OR 1.143 [1.036, 1.261]) were more susceptible than women (3d: OR 1.113 [1.036, 1.195]); older individuals (3d: OR 1.193 [1.058, 1.346]) were more susceptible than young and middle-ages (3d: OR 1.086 [1.015, 1.162]) and minors (3d: OR 1.101 [0.949, 1.276]). (At 1d, young and middle aged individuals were more susceptible than older individuals and minors.) Overweight patients (3d: OR 1.130 [1.020, 1.253]) were also more at risk than underweight (3d: OR 1.103 [0.910, 1.338]) and normal weight patients (3d: OR 1.112 [1.032, 1.198]). Statistical significance of group differences was not assessed. | 5 (high) |
| Shen et al. (2025) | Cohort study; logistic mixed effects models | 12,403 participants (35,211 observations) | General: individuals aged ≥45 years (from the China Health and Retirement Longitudinal Study) | China (2011-2018) | 90th, 92.5th, 95th percentile for 2-4 consecutive days (comparing daytime, nighttime, compound heatwave days) | Depressive symptoms measured using CESD-10 | Age (<65, ≥65 years); sex (male, female); education level (illiterate, elementary and below, middle school and above); residence (urban, rural). | N/A | For 3d compound heatwaves at the 95th percentile, individuals aged <65 and those with higher education were significantly more vulnerable. There were no significant differences reported for sex or residence (urban/rural) at this threshold. The results were largely consistent for daytime, nighttime, or compound heatwave days, though urban residents were at significantly higher risk for daytime heatwaves. | 5 (high) |
| Tang et al. (2021) | Time-series analysis with distributed lag non-linear model; subgroup analysis for age and sex. | 21,169 | Clinical: Schizophrenia hospitalisations during the warm season | Anhui Mental Health Center in Hefei, China (2005-2019) | Heat threshold (min. risk temperature) was 19·4°C. Introduced a new index of daily excess hourly heat (DEHH) above the heat threshold. | Schizophrenia hospitalisations obtained from electronic health records. | Sex (male, female); Age (≤40, >40) | N/A | There was a higher risk for men from lag0 (RR 1·059) to lag3 (RR 1·036), while no significant association between DEHH and schizophrenia was observed for women at any lag. There was also a higher risk for ages >40 from lag1 (RR 1·049) to lag3 (RR 1·039), while the association was not significant for younger ages. Statistical significance of group differences was not assessed. | 5 (high) |
| Thawonmas et al. (2024) | Time-stratified case-crossover study; distributed lag non-linear model | 1,049,592 | Clinical: suicide deaths | Japan (1973-2015) | ≥90th percentile | Suicide | Sex (male, female); age (0-64, ≥65) | N/A | Higher burden was reported for women and individuals aged ≥65 years. Statistical significance of group differences was not assessed. | 5 (high) |
| Thawonmas et al. (2025) | Time-stratified case-crossover study; distributed lag non-linear model | 8,472 | Clinical: suicide deaths | Chiang Mai & Bangkok, Thailand (2002-2021) | Hot temperatures: ≥67th percentile | Suicide | Sex (male, female); age (0-64, ≥65) | N/A | Significantly higher burden was reported for individuals aged <65. No significant sex difference was found. | 5 (high) |
| Ulrich et al. (2025) | Time-stratified case-crossover (matched) analysis using conditional quasi-Poisson models, stratified by subgroups (effect modification analysis) | 324,928 emergency department visits | Clinical: emergency department visits for psychiatric conditions in pregnant individuals | North Carolina, USA (May-September 2011-2019) | 95th percentile ≥3 days | Psychiatric admissions (primary or secondary ICD-10 codes) for perinatal mood and anxiety disorders (PMAD), severe mental illness (SMI), mental disorders of pregnancy (MDP), suicidality, and substance misuse | Age (<35, ≥35), race (Black, White, other), ethnicity (Hispanic, Non-Hispanic), insurance type (Private, Medicaid, self-pay, other), rural/urban commuting area (RUCA) (urban, rural, suburban), zipcode-level Index of Concentration at the Extremes for Race (ICE Race); zipcode-level ICE for Income (ICE Income) | N/A | In the effect modification analysis, significantly greater risk was found for pregnant individuals aged ≥35 years for SMI (RR 1.81 [1.45–2.25]); individuals with insurance type 'Other' for PMAD (RR 1.52 [1.25–1.85]), and individuals with Medicaid for SUB (RR 1.04 [1.00–1.08]); individuals with race 'Other' for SUB (RR 1.10 [1.04–1.16]); and those living in rural areas for SUB (RR 1.17 [1.02-1.33]). In contrast, significantly decreased risks were found for Black individuals for PMAD (RR 0.88 [0.85-0.92]); mixed-income neighbourhoods for PMAD (ICE Income Q3: RR 0.88 [0.82–0.95], low-income neighborhoods for SMI (ICE Income Q1: RR 0.84 [0.77–0.92]) and SUIT (RR 0.66 [0.60–0.72]; neighborhood racial segregation for PMAD (ICE Race Q1: RR 0.90 [0.84–0.95]), Medicaid for PMAD (RR 0.90 [0.87–0.93]) and insurance type ‘Other’ for SUIT (RR 0.65 [0.62–0.68]). | 5 (high) |
| Wang et al. (2018) | Time-series analysis using Poisson generalised linear regression model and distributed lag non-linear model Stratified analyses by age, sex, and marital status. | 17,744 | Clinical: Schizophrenia hospital admissions | Anhui Mental Health Centre, China (May-October 2005-2014) | 75th percentile of daily mean temperature (28°C) | Schizophrenia hospitalisations obtained from health records. | Sex (male, female); Age (0-20, 21-40, 41-60, >60); Marital status (married, unmarried) | N/A | There was a cumulatively higher risk for men (highest RR 1·12 at lag0-4). The age group at highest risk was 21-40 (highest RR 1·09 at lag0-4 and 0-5), followed by 41-60 (highest RR 1·11 at lag0-5). There was also increased cumulative risk for married patients (highest RR 1·07 at lag0-4 and 0-5). | 5 (high) |
| Wang, Hao, et al. (2025) | Longitudinal cohort study; logistic regression and multiplicative/additive interaction analyses, stratified by subgroups | 12,316 (including 5,807 new cases of depressive symptoms) | General: individuals aged ≥45 years (from the China Health and Retirement Longitudinal Study) | China (2013-2020) | 9 definitions of heatwave from 92.5th-97.5th percentile for 2-4 days; 3 days 95th percentile reported here as main analysis (34.85°C) | Depressive symptoms (CESD-10) | Age (<60, ≥60), gender (male, female), education level (low, high), city-level air pollution (PM2.5, PM10, CO and SO2) | City-level exposure to green (NDVI) and blue spaces | During heatwaves, lower level of green spaces (OR 1.58 [1.36-1.84]) or blue spaces (OR 1.31 [1.13-1.52]) was associated with an increased risk of depressive symptoms. For the heatwave-green space interaction, this effect was stronger among individuals <60 years, with no significant modification by gender or education. In contrast, higher air pollution (PM2.5, PM10, CO and SO2) increased risks of depressive symptoms during heatwaves. This effect was also pronounced in individuals <60 years. | 5 (high) |
| Wang, Li, et al. (2025) | Longitudinal cohort study using propensity score matching; ordered probit models and linear regression models; moderation and stratified analyses | 26,567 | General: older adults (>45 years) (from the Longitudinal Aging Study of India) | 10 states in India (2017-2018) | Severe heat exposure: At least 25 days in the month exceeding 32°C and highest temp over 47°C | Self-reported mental health: feeling depressed, tired, afraid, and satisfied with life; also self-reported depressive symptoms from CESD-10 |  | Presence of electricity supply; having water supply on site; ownership of cooling devices (further stratified by urban/rural residence) | Owning a cooling device mitigated the negative effect of heat on various mental health measures, including feeling depressed (β=-0.209, p<0.05), afraid (β=-0.375, p<0.01), and depressive symptoms measured by CESD (β=-1.005, p<0.01). This effect was most prominent in rural areas. The moderating effect of electricity supply as not significant. In rural areas, access to water on site was associated with higher likelihood of feeling depressed (β=0.273, p<0.01), tired (β=0.145, p<0.05), afraid (β=0.301, p<0.01), and higher CESD (β=1.034, p<0.01). In contrast, in urban areas, access to water on site was associated with a lower likelihood of feeling tired (β=-0.460, p<0.01). | 5 (high) |
| Yoo, Eum, Gao, et al. (2021) | Time-series analysis using distributed lag non-linear model and quasi-Poisson distribution; stratified analyses by risk factors | 94,636 | Clinical: Emergency room visits due to mental disorders | Erie and Niagara counties, New York State, USA (2009-2015) | 97·5th percentile (29°C) | Mental health-related emergency room visits obtained from New York Department of Health. | Age (0–19, 20–49, 50–64, > 64); Sex (male, female); Ethnicity (African-American, Caucasian-American, Other) | N/A | Cumulative risk was most pronounced in elderly and youth (RR: 1·59 and 1·48 at lag0-21, respectively), though significant effects were observed for ages 20-49 and 50-64 for shorter lag periods. There was no significant modification effect by sex or by ethnicity. | 5 (high) |
| Yoo, Eum, Roberts, et al. (2021) | Time-series analysis using a quasi-Poisson generalised linear model and distributed lag non-linear model. Meta-analysis to pool risk estimates across different regions and stratified analyses. | 2,893,794 | Clinical: Emergency room visits due to mental disorders | New York State, USA (2009-2016) | 97·5th percentile (27·07°C) | Mental health-related emergency room visits obtained from New York Department of Health. | Age (0–19, 20–49, 50–64, > 64); Sex (male, female); Ethnicity (White, Black, Hispanic, Asian, Other) | N/A | There were no significant differences reported for age, sex, or ethnicity over the lag period. | 5 (high) |
| Zhang et al. (2024) | Time-series study; quasi-Poisson generalised linear models, stratified by subgroups (modification analysis) | 575,505 outpatient visits | Clinical: outpatient visits for mental disorders | Guangzhou, China (May-October 2010-2014) | 9 different heatwave criteria: 90th, 92.5th and 95th percentiles of daily mean temperature over 2-4 days | Daily outpatient visits for mental disorders (ICD-10) | Age (<60, ≥60); gender (male, female); health insurance type ('Urban Employee‐based Basic Medical Insurance'/UEBMI for urban employees or retirees; 'Urban Resident‐based Basic Medical Insurance'/URBMI for non-working urban residents) | N/A | Only one heatwave definition (≥2 days 95th percentile) showed a significant difference between subgroups, where those aged >60 (RR 1.323 [1.095,1.559]) had higher risk. All other subgroup analyses were nonsignificant. | 5 (high) |
| Zhong et al. (2025) | Time-stratified case-crossover study; conditional logistic regression with distributed lag non-linear models, stratified by subgroups (modification analysis) | 762,895 visits | Clinical: outpatients seeking treatment for mental disorders | Anhui Province, China (2019-2021) | 90th percentile | Outpatient visits for mental disorders (incl. schizophrenia, depression, anxiety) (ICD-10) | Age (<18, 18–34, 35–49, 50–64, ≥65 years old); sex (male, female). | N/A | Women were at higher risk for depression (OR 1.19 [1.08–1.30]) or anxiety (OR 1.20 [1.08–1.35]), while men were more likely to experience schizophrenia (OR 1.21 [1.09–1.35]). Furthermore, for schizophrenia and anxiety, those aged ≥35 were at higher risk, while for depression, those aged <35 were at higher risk. Statistical significance of group differences was not assessed. | 5 (high) |
| Zhou et al. (2023) | Time-series analysis using quasi-Poisson generalised linear model and distributed lag non-linear model; hierarchical analyses for age and sex. | 155,436 | Clinical: Outpatient visits by patients with depression | 9 urban districts in Chongqing, China (2014-2019) | Extreme high humidex (temperature + humidity): value over 40 (great discomfort; e.g., temperature of 30°C and humidity of 70% would be 41 humidex) | Depression outpatient visits obtained from hospital records. | Sex (male, female); Age (≤18, 19-59, ≥60) | N/A | Women were more vulnerable (RR significant at lags 1, 2, 12) with attributable fraction 1·20% compared to 0·92% for males. Ages >60 were also more vulnerable (RR significant at lags 0, 1, 2, 8, 9, 10, 11, 12) with attributable fraction 2·24% compared to 1·02% in 19-59 years. Statistical significance of subgroup differences was not assessed. | 5 (high) |
| Zhou et al. (2024) | Time-series analysis; Poisson generalised linear model with distributed lag non-linear models, stratified by subgroups | 10,420 admissions | Clinical: hospital admissions for schizophrenia | Liuzhou, China (2013-2020) | 75th percentile of daily mean temperature (21.65°C) | Hospital admission for schizophrenia (ICD-10) | Gender (male, female), age (0-20, 21-40, 41-60, ≥61), marital status (married, unmarried) | N/A | For gender, the single lag effects were observed for both genders and were greatest in women on lag4 (RR 1.075 [1.006–1.148]) and in men on lag5 (RR 1.068 [1.004–1.136]). The cumulative lag effects were nonsignificant in women, but were significant for men at lag0-1, lag0-4, and lag0-7 with a maximum effect at the latter (RR 1.414 [1.16–1.724]). For age, single lag effects were significant at some lag days for all ages <61. For 0-20, the greatest effect was at lag6 (RR 1.125 [1.015–1.247]), for 21-40 at lag5 (RR 1.087 [1.004–1.177]), and for 41-60 at lag5 (RR 1.087 [1.004–1.177]). Cumulative lag effects were only reported for those aged 21–40 from lag0–3 to lag0–7, with the greatest effect at lag0–7 (RR 1.401 [1.148–1.708]). For marital status, the effects were higher in unmarried individuals for most single and cumulative lag effects, while all lag effects for married individuals were nonsignificant. | 5 (high) |

## S8. Further details on Table 2 which can be found in the main manuscript.

Overview of the qualitative and mixed-method studies included in the systematic review. A summary table was provided in the main manuscript (Table 2). If a study reported on different types of climate events or climate change in general, only findings related to heat events were extracted. This table includes an overall quality score for each study; an item-by-item breakdown of this score is provided in the supplementary materials above (S3).

*Abbreviations: N/A, not applicable.*

| Study | Study design and analysis | N | Population type | Location and study period | Definition of heat | Mental health outcome | Risk factors investigated | Protective factors investigated | Findings | Quality score (scale 1-5) |
| --- | --- | --- | --- | --- | --- | --- | --- | --- | --- | --- |
| Goudet et al. (2024) | 55 semi-structured interviews and 6 focus groups. Used a reflexive ethnography approach and thematic framework analysis based on a social determinants framework. | 80 | General: Communities vulnerable to climate events during the hot and monsoon seasons. | Bangladesh (March, April, October 2022) | Not specified | Self-reports of climate-related mental health impact in women in communities vulnerable to climate events. | Caring responsibilities | N/A | Participants described that children's heat-induced sleep problems lead to more behavioural issues during the day, which was reported to increase anxiety and stress. | 2 (Low) |
| Hossain et al. (2024) | Mixed methods: Cross-sectional surveys (chi-square tests for sociodemographic subgroups); qualitative focus groups and interviews (thematic analysis) | Surveys (310 households); 4 focus groups (n=7-10 each); key informant interviews (n=10) | General: residents in urban households | Rajshahi, Bangladesh (time period not specified) | Heatwave day >38°C, though not specified in surveys, focus groups or interview. | Self-reported mental health impact, including anxiety (42%), irritability (28%), and sleep disturbance (36%) | Quantitative: sex, education level; Qualitative: age, sex, caregiving responsibilities | N/A | Women experienced higher anxiety levels (exacerbated by caregiving responsibilities), as did those without formal education. Older people reported more sleep disturbances. | 2 (Low) |
| Kadio et al. (2024) | Qualitative: in-depth individual interviews, focus group discussions, and key informant interviews; analysed using inductive thematic analysis | 40 interviews (10 pregnant and 10 postpartum in each of the 2 sites), 12 in focus groups (2 women of reproductive age, 2 mothers-in-law, and 2 male spouses for each site) and 19 key informant interviews (9 Bodogo and 10 Kaya) | General: pregnant and postpartum women accessing health facilities; women of reproductive age; mothers-in-law; male spouses; health workers; community representatives | Bogodogo (urban) and Kaya (rural), Burkina Faso (March-October 2020) | Not specified but region experiences heat reaching 40•C for several days | Not specified but asked about wellbeing and functioning (including social and psychological consequences) during pregnancy and postpartum periods | Pregnancy, postpartum period, caring responsibilities | N/A | Pregnant and postpartum women were reported to experience sadness, anxiety, irritability, anger and stress during heat. Reasons included the physical discomfort of being pregnant, seeing children suffering from the heat, having to stay indoors, and challenges feeding babies. | 2 (Low) |
| Palinkas et al. (2022) | 40 semi-structured qualitative interviews. Analysed using thematic content analysis. | 40 | General: Low-income residents of urban heat islands who are parents of students participating in college prep programme. | Los Angeles, California, USA (November 2021-January 2022) | Not specified | Self-reported heatwave impacts on own or children's physical or mental health | Caring responsibilities | N/A | 13 participants reported concerns about their children's health during heatwaves and that this can cause them to worry, e.g., staying hydrated and cool. 29 participants reported mental health effects of heatwaves, with 7 participants reporting health effects due to concern for children. | 1 (Low) |
| Pardon et al. (2024) | 7 semi-structured focus groups. Analysed using reflexive thematic analysis. | 31 | General: Mothers (min. age 18) of children under 12 months. | Queensland, Australia (February-April 2023) | Not specified | Self-reported maternal mental health and wellbeing | Caring responsibilities | N/A | Mothers reported increased worry about their child(ren)'s safety and wellbeing during heat, specifically regarding sufficient hydration, body temperature, sickness, "mom guilt" over activity constraints due to avoiding outdoors, feelings of frustration and discomfort, not being able to get the usual benefits from nature, and increased social isolation due to staying at home. | 3 (Medium) |

## S9. Demographics of focus group participants.

Data on gender, age, history of mental illness, and ethnicity were self-reported by the participants. Information on type of diagnosis or treatment history was not collected.

**Data missing for one participant who selected ‘Prefer not to say’.*

***Data missing for two participants who selected ‘Prefer not to say’.*

****Nine of the participants in the healthcare group directly worked in the field of mental health with the following job roles: psychotherapist, art psychotherapist, psychologist, psychiatrist, clinical psychologist, trainee clinical psychologist (n=2), substance misuse worker, support worker. The three healthcare professionals who were not mental health professionals (nurse, healthcare assistant, occupational therapist) had experience of supporting patients with mental health-related issues as part of their practice.*

*Abbreviations: N/A, not applicable.*

|  |  | Participants with lived experience | | | | Healthcare professionals | | | |
| --- | --- | --- | --- | --- | --- | --- | --- | --- | --- |
|  |  | Group 1 | Group 2 | Group 3 | Total | Group 1 | Group 2 | Group 3 | Total |
| N |  | 8 | 6 | 7 | 21 | 3 | 4 | 5 | 12 |
| Gender (N, %) | Male | 1 (12.5%) | 1 (16.7%) | 3 (42.9%) | 5  (23.8%) | 0  (0.0%) | 0  (0.0%) | 0  (0.0%) | 0  (0.0%) |
|  | Female | 7 (87.5%) | 5 (83.3%) | 4 (57.1%) | 16 (76.2%) | 2 (66.7%) | 4 (100.0%) | 5 (100.0%) | 11 (91.7%) |
|  | Nonbinary | 0  (0.0%) | 0  (0.0%) | 0  (0.0%) | 0  (0.0%) | 1 (33.3%) | 0  (0.0%) | 0  (0.0%) | 1  (8.3%) |
| Mean age (age range) |  | 24.6  (20-36) | 27.3  (22-36)* | 41.9  (22-62) | 31.5  (20-62)* | 44.3  (26-56) | 26.0  (23-29)** | 31.6  (28-39) | 33.5 (23-56)** |
| % with current or past mental illness |  | 62.50% | 83.30% | 71.40% | 68.20% | 0% | 25.00% | 20.00% | 16.70% |
| % working in mental health care*** |  | N/A | N/A | N/A | N/A | 66.70% | 50.00% | 100.00% | 75.00% |
| Ethnicity (N, %) | Caucasian | 1 (12.5%) | 3 (50.0%) | 2 (28.6%) | 6  (28.6%) | 2 (66.7%) | 2  (50.0%) | 3  (60.0%) | 7  (58.3%) |
|  | Asian | 5 (62.5%) | 1 (16.7%) | 4 (57.1%) | 10 (47.6%) | 0  (0.0%) | 1  (25.0%) | 1  (20.0%) | 2  (16.7%) |
|  | Black | 0 (0.0%) | 0 (0.0%) | 0 (0.0%) | 0 (0.0%) | 0 (0.0%) | 1 (25.0%) | 0 (0.0%) | 1 (8.3%) |
|  | Other | 1* (12.5%) | 1* (16.7%) | 1 (14.3%) | 3** (14.3%) | 1 (33.3%) | 0  (0.0%) | 1  (20.0%) | 2  (16.7%) |
| Mode of focus group |  | In-person | Virtual | Virtual | N/A | Virtual | Virtual | Virtual | N/A |

## S10. Visual overview of the results of the joint thematic content analysis for risk and protective factors.

S11. Results of the joint thematic content analysis for risk and protective factors with supportive quotes from participants.

A visual overview on themes and subthemes is provided above in S10.

| Themes | Sub-themes | *Participant quotes* |
| --- | --- | --- |
| Ability to adapt behaviour | **Mental/cognitive inability to adapt behaviour** (e.g., due to learning disability, mental health condition) | *"A big risk factor would be those with symptom processing difficulties or lack of interoceptive awareness, because I think those two fundamentally put people at risk to either be too aware, i.e., they're just feeling like they cannot even function in the heat and really, sort of feeling the effect of the heat very negatively, or those that are a little bit less aware and actually are maybe going out for runs or doing sort of behaviours that aren't beneficial in that temperature and then putting themselves at risk. So, I think it's just like people maybe you have hypochondriacs, but also people that have just got a sort of slight difficulty in identifying their body needs and the interoceptive awareness." (Healthcare professional)* |
|  | **Physical or external constraints** (e.g., impaired mobility, inflexible schedule, lack of financial means) | *“If you got a bit more money, you can buy fans. You can buy cooling things, whatever you need. Ice creams, whatever. […] If you’re not as financially well off, you haven't got that extra reserve. You might not be able to afford the things that can help you cool down. So you're just kind of stuck with the heat, which can make you feel a lot more stressed than a person who has got more to help them out with it.” (Person with lived experience)* |
|  | **Social support system** (e.g., social isolation, lack of knowledge of coping mechanisms) | *"Online is a really great alternative to still checking in, still doing a zoom, doing a Teams. So you're getting that social – ideal is person-to-person, but at least there is still some consistency and there's a lack of isolation because then you're saying, 'Oh my goodness, it’s so hot', 'me as well' and you kind of feel heard and validated." (Healthcare professional)* |
| Personal heat sensitivity | **Altered thermoregulation** (e.g., medication, physical health condition, menopause, pregnancy, substance use) | *"I feel that with different [physical health] conditions there will be different reactions and outcomes to their reaction towards heat. [...] So like in my case [of having gone through chemotherapy], like I said specifically, I believe is you know over like the radiation everything with the more inflammation within my physiology, hence the lower threshold towards heat." (Person with lived experience)* |
|  | **Resilience to external stressors** (e.g., easily overwhelmed, caring responsibilities) | *“I think where somebody's [mental well-being] starting point from as well is has a huge impact. So, my mental health is okay, so it means that if it's really hot, I'm able to do something to help myself […]. But if I've got some kind of mental health issue, […] then it might mean that my resilience is affected and my resourcefulness will be affected as well.” (Healthcare professional)* |
| Disparities in heat exposure | **Living environment** (e.g., homelessness; housing that tends to run hot) | *“I usually spend summer at my parents’ house and I was thinking the other day that if I was not at my parents' house at the most hottest days of the year, I would probably struggle way more if I was like in a studio apartment in the 10th floor somewhere.” (Person with lived experience)* |
|  | **Working environment** (e.g., physically hot and/or straining work environment) | *"My sister's work didn't have anything to cool the building and she said it was so stressful and she came away from work stressed in a way that was unusual for her because she wasn't working in the right conditions. But because I don't think the laws in the UK, if there’s if a building gets below a certain temperature, I believe that there are rules about having to not be in that workspace, but above a certain temperature. I feel like there's something missing there in the UK. And they were working in an office, but I think it was about 40 something degrees it was, it was crazy, and all of them were so upset that they couldn't do anything about it. So I think working conditions is a big one - or appropriate working conditions for the temperatures is a big one." (Person with lived experience)* |
|  | **Leisure** (e.g., activities associated with heat exposure, like exercise outdoors) | *"What I would add is another question to this set of questions on the basis of whether what kind of job the patient could have, whether it involves standing for long hours outside in the sun or whether he or she would be exposed to weather conditions or if they might have — not only when it comes to a job, perhaps they have a hobby they love. I don't know. Hiking in the countryside, you know? So that's would also be a major risk factor, in my opinion." (Person with lived experience)* |
|  | **Access to cool spaces** (e.g., green/blue spaces, artificial spaces) | *"I'm thinking of my workplace for example. It is quite an escape for me when it gets very hot because there is actual air conditioning, so on hot days when I have to got to work once I've travelled through — as probably many of you know, transport in London is generally very hot, very inappropriate for the weather — so once I've got to work, I really feel like I can finally breathe and calm down and gather myself." (Person with lived experience)* |

S12. Supportive quotes from participants for each question in HEAT-MH.

Full details on HEAT-MH and supportive quotes from the co-development process in the focus groups were provided in the main manuscript (Table 3).

*Abbreviations: HCP, healthcare professional*

| Screening tool questions | | Quotes about the question content |
| --- | --- | --- |
| *Section 1: Prior experiences* | | |
| 1 | Compared to those around you, do you feel more susceptible to temperature changes in your environment or less able to respond to heat? For example, this could mean that you sweat more or feel easily overwhelmed in the heat. | *"Certain things irritate me more than other people would. [...] Sometimes I don't like to have my hair open because it sticks in my face. And maybe some people will have that with having it tied up and then when it's really hot you can’t do certain things - some people might get overstimulated. If you're sweating and then your legs stick together, some people get more irritated than others by things like that." (Person with lived experience)* |
| 2 | On days of hot weather, do you tend to change your behaviour or daily routine? For example, you might dress differently or not go outside as much. | *"Those that are a little bit less aware and actually are maybe going out for runs or doing sort of behaviours that aren't beneficial in that temperature and then putting themselves at risk." (HCP)* |
| *Section 2: General health* | | |
| 3 | Are you older than 65 years?* | *"I think younger people generally are better at managing heat. Whereas older people - the body being not as young - will struggle a bit more." (Person with lived experience)* |
| 4 | Do you have a long-term physical health condition? | *"I feel that with different conditions there will be different reactions and outcomes to their reaction towards heat. [...] In my case [having gone through chemotherapy], […] I believe is over like the radiation, everything with the more inflammation within my physiology, hence the lower threshold towards heat." (Person with lived experience)* |
| 5 | Do you have an existing mental health diagnosis? | *“If there are individuals who have an existing mental health problem or a predisposition to it, and if and when they experience an episode of crisis […], are they able to recognise [..] that extreme heat they're experiencing is contributing to it rather than some other unrelated cause? […] If they don't recognise it, they may not be able to take precautionary measures and address the issue.“ (Person with lived experience)* |
| 6 | Is your mobility limited in any way? For example, if it gets hot in a room you are in, are you able to look for a cooler space by yourself? | *"About mobility as well, like being able to escape the heat is a big one. I'm just thinking of the house that I used to live in. Upstairs used to be really hot and maybe if someone isn't able to get down the stairs by themselves - there's no one at home to help them down - maybe they're then trapped in a room upstairs that's hotter than downstairs." (HCP)* |
| 7 | Are you currently taking any of the medications included on this list: Heart medication, Antidepressants, Benzodiazepines, Antipsychotics, Dopaminergics, Antihistamines, Anticholinergics, Decongestants, Stimulants, Anti-seizure medication/antiepileptics? | *"I take quite a bit of medication for my mental health, for my diabetes and [...] a broken spine [...]. Side effects of the medication is that it will make my body produce heat as well and on top of that, if there's extreme heat, [...] all of the symptoms that I have just get compounded." (Person with lived experience)* |
| 8 | Are you currently pregnant or suspect you might be? | *"I was just working in perinatal mental health and just that period of post-natal period and with like the lack of sleep anyway. And I was just really thinking of, like sleep and anxiety and the hypervigilance that people have after giving birth." (HCP)* |
| 9 | Are you currently going through menopause or have you noticed any symptoms that could be linked to perimenopause? Symptoms include, but are not limited to, irregular periods, hot flashes, night sweats, or mood changes. | *“I know we've mentioned age, the other thing I was quite mindful of is older women around hormonal changes and there's a lot more that people are becoming more aware of around menopause and those age, you know, age-related factors which is very individualistic.” (HCP)* |
| *Section 3: Daily life* | | |
| 10 | Do you currently lack access to shelter that can protect you from weather exposure, such as sleeping outdoors, in a tent, or in a car? | *"My homeless clients are really vulnerable. One of my clients lives in a tent on a roundabout. [...] He also finds it very difficult to stay either warm or to stay cool, depending on what the weather is, and living in a tent will kind of intensify heat as well." (HCP)* |
| 11 | Does your housing tend to get uncomfortably hot during hot weather without access to cooler spaces? | *“I just moved into this new flat and this one is better in terms of like when it's hot outside at keeping the inside a bit cooler, but the previous room I lived in was just an absolute sauna. […] My previous place was just a room […], this is a whole flat. So, that means that if one room gets a bit hotter, you can move to another one and you still your space that you can use.” (Person with lived experience)* |
| 12 | During hot weather, do you feel you have little or no support from others to help you stay cool and safe? | *“I wonder whether the social isolation for maybe populations of people who live alone already, like during COVID, there were people, old people, that were just alone for extended periods of time, especially as being elderly puts you at risk in itself, and then you are alone as well, that I feel like they go together a bit.” (Person with lived experience)* |
| 13 | Are you a carer for someone? This may include children, elderly adults, or someone living with a mental illness. | *“Once you become a parent, you have responsibility in a sense that that might play into it, and that might be a bit of a risk factor for your own mental health. If you know that you're not just worried about your own physical mental health, you're worried about the physical mental health of those around you.“ (Person with lived experience)* |
| 14 | If you work, is it difficult to keep cool at your workplace during hot weather? For example, you might work outside or around hot ovens. | *"I think certain environments might be quite difficult to keep cool, so I'm thinking about like a chef in a kitchen, got lots of ovens on, it's very difficult to keep cool." (HCP)* |
| 15 | Do you regularly drink more than 14 units of alcohol per week during periods of hot weather?* One pint of lower strength lager/beer/cider is 2 units; one pint of higher strength beer is 3 units; one standard glass of wine is 2.1 units. | *"Particularly, for me, my client group who drink a lot of alcohol. It is quite a big risk in terms of their physical health, but also hydration again, which does affect their mental health and their capacity to stay safe." (HCP)* |

## References

Baecker, L., Iyengar, U., Del Piccolo, M. C., & Mechelli, A. (2025). Impacts of extreme heat on mental health: Systematic review and qualitative investigation of the underpinning mechanisms. *The Journal of Climate Change and Health*, *22*, 100446. https://doi.org/10.1016/j.joclim.2025.100446

Bao, Y., Li, Y., Gu, J., Shen, C., Zhang, Y., Deng, X., Han, L., & Ran, J. (2025). Urban heat island impacts on mental health in middle-aged and older adults. *Environment International*, *199*, 109470. https://doi.org/10.1016/j.envint.2025.109470

Basu, R., Gavin, L., Pearson, D., Ebisu, K., & Malig, B. (2018). Examining the Association Between Apparent Temperature and Mental Health-Related Emergency Room Visits in California. *American Journal of Epidemiology*, *187*(4), 726–735. https://doi.org/10.1093/aje/kwx295

Bundo, M., De Schrijver, E., Federspiel, A., Toreti, A., Xoplaki, E., Luterbacher, J., Franco, O. H., Müller, T., & Vicedo-Cabrera, A. M. (2021). Ambient temperature and mental health hospitalizations in Bern, Switzerland: A 45-year time-series study. *PLOS ONE*, *16*(10), e0258302. https://doi.org/10.1371/journal.pone.0258302

Bundo, M., Preisig, M., Merikangas, K., Glaus, J., Vaucher, J., Waeber, G., Marques-Vidal, P., Strippoli, M.-P. F., Müller, T., Franco, O., & Vicedo-Cabrera, A. M. (2023). How ambient temperature affects mood: An ecological momentary assessment study in Switzerland. *Environmental Health*, *22*(1), 52. https://doi.org/10.1186/s12940-023-01003-9

Cohen, G., Rowland, S. T., Benavides, J., Lindert, J., Kioumourtzoglou, M.-A., & Parks, R. M. (2024). Daily temperature variability and mental health-related hospital visits in New York State. *Environmental Research*, *257*, 119238. https://doi.org/10.1016/j.envres.2024.119238

Corvetto, J. F., Federspiel, A., Sewe, M. O., Müller, T., Bunker, A., & Sauerborn, R. (2023). Impact of heat on mental health emergency visits: A time series study from all public emergency centres, in Curitiba, Brazil. *BMJ Open*, *13*(12), e079049. https://doi.org/10.1136/bmjopen-2023-079049

Corvetto, J. F., Helou, A. Y., Kriit, H. K., Federspiel, A., Bunker, A., Liyanage, P., Costa, L. F., Müller, T., & Sauerborn, R. (2024). Private vs. public emergency visits for mental health due to heat: An indirect socioeconomic assessment of heat vulnerability and healthcare access, in Curitiba, Brazil. *Science of The Total Environment*, *934*, 173312. https://doi.org/10.1016/j.scitotenv.2024.173312

Crank, P. J., Hondula, D. M., & Sailor, D. J. (2023). Mental health and air temperature: Attributable risk analysis for schizophrenia hospital admissions in arid urban climates. *Science of The Total Environment*, *862*, 160599. https://doi.org/10.1016/j.scitotenv.2022.160599

Critical Appraisal Skills Programme. (2024). *CASP Checklist: For Qualitative Research*. CASP UK.

Dang, T. N., Vy, N. T. T., Thuong, D. T. H., Phung, D., Van Dung, D., & Le An, P. (2022). Main and added effects of heatwaves on hospitalizations for mental and behavioral disorders in a tropical megacity of Vietnam. *Environmental Science and Pollution Research*, *29*(39), 59094–59103. https://doi.org/10.1007/s11356-022-19898-1

Dey, C., Wu, J., Uesi, J., Sara, G., Dudley, M., Knight, K., Scott, J. G., Jay, O., Bowden, M., & Perkes, I. E. (2025). Youth suicidality risk relative to ambient temperature and heatwaves across climate zones: A time series analysis of emergency department presentations in New South Wales, Australia. *Australian & New Zealand Journal of Psychiatry*, *59*(1), 18–28. https://doi.org/10.1177/00048674241290449

Dumont, C. R., & Mathis, W. S. (2023). Mapping Heat Vulnerability of a Community Mental Health Center Population. *Community Mental Health Journal*, *59*(7), 1330–1340. https://doi.org/10.1007/s10597-023-01119-9

Fang, B., & Zhang, Q. (2025). Heatwaves and its impact on the depressive symptoms among Chinese community-dwelling older adults: Examining the role of social participation. *Archives of Gerontology and Geriatrics*, *129*, 105668. https://doi.org/10.1016/j.archger.2024.105668

Florido Ngu, F., Kelman, I., Chambers, J., & Ayeb-Karlsson, S. (2021). Correlating heatwaves and relative humidity with suicide (fatal intentional self-harm). *Scientific Reports*, *11*(1), 22175. https://doi.org/10.1038/s41598-021-01448-3

Gao, X., Liu, S., Jing, X., Wang, R., Song, M., Liu, X., Wang, X., Wang, L., & An, C. (2023). Protective effects of low temperature on the risk of hospitalization for mental and behavioral disorders: A time series study. *Postgraduate Medicine*, *135*(5), 493–500. https://doi.org/10.1080/00325481.2023.2201148

Goudet, J.-M., Binte Arif, F., Owais, H., Uddin Ahmed, H., & Ridde, V. (2024). Climate change and women’s mental health in two vulnerable communities of Bangladesh: An ethnographic study. *PLOS Global Public Health*, *4*(6), e0002080. https://doi.org/10.1371/journal.pgph.0002080

Green, J., & Thorogood, N. (2018). *Qualitative methods for health research* (4th edition). SAGE.

Guo, M., Zheng, J., Zhang, J., Li, J., Wang, Q., & Lai, D. W. L. (2025). Impact of extreme weather events on mental health among older adults in China: A longitudinal study. *Sustainable Cities and Society*, *130*, 106580. https://doi.org/10.1016/j.scs.2025.106580

Hansen, A., Bi, P., Nitschke, M., Ryan, P., Pisaniello, D., & Tucket, G. (2008). The Effect of Heat Waves on Mental Health in a Temperate Australian City. *Environmental Health Perspectives*, *116*(10), 1369–1375. https://doi.org/doi:10.1289/ehp.11339

Hossain, I., Rana, Md. S., Haque, A. K. M. M., & Al Masud, A. (2024). Urban household adaptation to extreme heatwaves: Health impacts, socio-economic disparities and sustainable strategies in Rajshahi. *Discover Sustainability*, *5*(1), 518. https://doi.org/10.1007/s43621-024-00697-2

Hu, J., Hu, W., Xu, Z., Peng, C., Cheng, J., Rong, F., Wang, Y., Zhang, N., Guan, M., & Yu, Y. (2025). Associations of exposure to heatwaves with depression and anxiety among adolescents: A cross-sectional study of the Chinese adolescent health survey. *Journal of Affective Disorders*, *387*, 119499. https://doi.org/10.1016/j.jad.2025.119499

Huebner, G. M. (2022). The role of parenthood in worry about overheating in homes in the UK and the US and implications for energy use: An online survey study. *PLOS ONE*, *17*(12), e0277286. https://doi.org/10.1371/journal.pone.0277286

Kadio, K., Filippi, V., Congo, M., Scorgie, F., Roos, N., Lusambili, A., Nakstad, B., Kovats, S., & Kouanda, S. (2024). Extreme heat, pregnancy and women’s well-being in Burkina Faso: An ethnographical study. *BMJ Global Health*, *8*(Suppl 3), e014230. https://doi.org/10.1136/bmjgh-2023-014230

Lavigne, E., Maltby, A., Côté, J.-N., Weinberger, K. R., Hebbern, C., Vicedo-Cabrera, A. M., & Wilk, P. (2023). The effect modification of extreme temperatures on mental and behavior disorders by environmental factors and individual-level characteristics in Canada. *Environmental Research*, *219*, 114999. https://doi.org/10.1016/j.envres.2022.114999

Lee, S., Lee, H., Myung, W., Kim, E. J., & Kim, H. (2018). Mental disease-related emergency admissions attributable to hot temperatures. *Science of The Total Environment*, *616–617*, 688–694. https://doi.org/10.1016/j.scitotenv.2017.10.260

Li, K., Henderson, S. B., Coker, E. S., McLean, K. E., & Lee, M. J. (2025). The association between hot days and substance-related suicides: A time-stratified case-crossover analysis in British Columbia, Canada. *Environmental Health*, *24*(1), 25. https://doi.org/10.1186/s12940-025-01176-5

Liu, J., Yu, W., Pan, R., He, Y., Wu, Y., Yan, S., Yi, W., Li, X., Song, R., Yuan, J., Liu, L., Wei, N., Jin, X., Li, Y., Liang, Y., Sun, X., Mei, L., Song, J., Cheng, J., & Su, H. (2022). Association between sequential extreme precipitation-heatwaves events and hospitalizations for schizophrenia: The damage amplification effects of sequential extremes. *Environmental Research*, *214*, 114143. https://doi.org/10.1016/j.envres.2022.114143

Liu, X., Liu, H., Fan, H., Liu, Y., & Ding, G. (2018). Influence of Heat Waves on Daily Hospital Visits for Mental Illness in Jinan, China—A Case-Crossover Study. *International Journal of Environmental Research and Public Health*, *16*(1), 87. https://doi.org/10.3390/ijerph16010087

Mahakalkar, A. U., Gianquintieri, L., Amici, L., Brovelli, M. A., & Caiani, E. G. (2024). Geospatial analysis of short-term exposure to air pollution and risk of cardiovascular diseases and mortality–A systematic review. *Chemosphere*, *353*, 141495. https://doi.org/10.1016/j.chemosphere.2024.141495

Mason, L. R., Erwin, J., Brown, A., Ellis, K. N., & Hathaway, J. M. (2018). Health Impacts of Extreme Weather Events: Exploring Protective Factors with a Capitals Framework. *Journal of Evidence-Informed Social Work*, *15*(5), 579–593. https://doi.org/10.1080/23761407.2018.1502115

Mason, L. R., Sharma, B. B., Walters, J. E., & Ekenga, C. C. (2020). Mental Health and Weather Extremes in a Southeastern U.S. City: Exploring Group Differences by Race. *International Journal of Environmental Research and Public Health*, *17*(10), 3411. https://doi.org/10.3390/ijerph17103411

Min, M., Shi, T., Ye, P., Wang, Y., Yao, Z., Tian, S., Zhang, Y., Liang, M., Qu, G., Bi, P., Duan, L., & Sun, Y. (2019). Effect of apparent temperature on daily emergency admissions for mental and behavioral disorders in Yancheng, China: A time-series study. *Environmental Health*, *18*(1), 98. https://doi.org/10.1186/s12940-019-0543-x

Nitschke, M., Tucker, G. R., & Bi, P. (2007). Morbidity and mortality during heatwaves in metropolitan Adelaide. *Medical Journal of Australia*, *187*(11–12), 662–665. https://doi.org/10.5694/j.1326-5377.2007.tb01466.x

Niu, L., Girma, B., Liu, B., Schinasi, L. H., Clougherty, J. E., & Sheffield, P. (2023). Temperature and mental health–related emergency department and hospital encounters among children, adolescents and young adults. *Epidemiology and Psychiatric Sciences*, *32*, e22. https://doi.org/10.1017/S2045796023000161

Nori-Sarma, A., Sun, S., Sun, Y., Spangler, K. R., Oblath, R., Galea, S., Gradus, J. L., & Wellenius, G. A. (2022). Association Between Ambient Heat and Risk of Emergency Department Visits for Mental Health Among US Adults, 2010 to 2019. *JAMA Psychiatry*, *79*(4), 341. https://doi.org/10.1001/jamapsychiatry.2021.4369

Pailler, S., & Tsaneva, M. (2018). The effects of climate variability on psychological well-being in India. *World Development*, *106*, 15–26. https://doi.org/10.1016/j.worlddev.2018.01.002

Palinkas, L. A., Hurlburt, M. S., Fernandez, C., De Leon, J., Yu, K., Salinas, E., Garcia, E., Johnston, J., Rahman, Md. M., Silva, S. J., & McConnell, R. S. (2022). Vulnerable, Resilient, or Both? A Qualitative Study of Adaptation Resources and Behaviors to Heat Waves and Health Outcomes of Low-Income Residents of Urban Heat Islands. *International Journal of Environmental Research and Public Health*, *19*(17), 11090. https://doi.org/10.3390/ijerph191711090

Pardon, M. K., Dimmock, J., Chande, R., Kondracki, A., Reddick, B., Davis, A., Athan, A., Buoli, M., & Barkin, J. L. (2024). Mental health impacts of climate change and extreme weather events on mothers. *European Journal of Psychotraumatology*, *15*(1), 2296818. https://doi.org/10.1080/20008066.2023.2296818

Park, J., Kim, A., Bell, M. L., Kim, H., & Lee, W. (2024). Heat and hospital admission via the emergency department for people with intellectual disability, autism, and mental disorders in South Korea: A nationwide, time-stratified, case-crossover study. *The Lancet Psychiatry*, *11*(5), 359–367. https://doi.org/10.1016/s2215-0366(24)00067-1

Park, J., Moon, J., Kwon, D., Ji, J. S., Kim, H., & Kim, Y. (2024). A protective role of urban greenspace on the association between night-time heat and suicide in Seoul, South Korea. *Environmental Research: Health*, *2*(1), 015005. https://doi.org/10.1088/2752-5309/ad1c42

Parks, R. M., Rowland, S. T., Do, V., Boehme, A. K., Dominici, F., Hart, C. L., & Kioumourtzoglou, M.-A. (2023). The association between temperature and alcohol- and substance-related disorder hospital visits in New York State. *Communications Medicine*, *3*(1), 118. https://doi.org/10.1038/s43856-023-00346-1

Runkle, J. D., Sugg, M. M., Berry, A., Reed, C., Cowan, K., Wertis, L., & Ryan, S. (2024). Association of Psychiatric Emergency Visits and Warm Ambient Temperature during Pregnancy: A Time-Stratified Case-Crossover Study. *Environmental Health Perspectives*, *132*(6), 067001. https://doi.org/10.1289/EHP13293

Schulte, F., Röösli, M., & Ragettli, M. S. (2024). Risk, Attributable Fraction and Attributable Number of Cause-Specific Heat-Related Emergency Hospital Admissions in Switzerland. *International Journal of Public Health*, *69*. https://doi.org/10.3389/ijph.2024.1607349

Shang, J., Xu, J., Xie, T., Ji, J. S., Tang, X., Wang, J., Wang, T., Liu, Y., Zhu, D., & Huang, C. (2025). The mediating role of thyroid hormone in the association between heatwave exposure and depression severity. *Environment International*, *200*, 109523. https://doi.org/10.1016/j.envint.2025.109523

Shen, J., Zhang, Y., Yuan, Y., Zhang, Y., & Hu, C. (2025). Daytime, nighttime, and day-night compound heatwaves and the risk of depression: A Chinese nationwide cohort. *Environmental Pollution*, *384*, 126913. https://doi.org/10.1016/j.envpol.2025.126913

Sung, T.-I., Chen, M.-J., & Su, H.-J. (2013). A positive relationship between ambient temperature and bipolar disorder identified using a national cohort of psychiatric inpatients. *Social Psychiatry and Psychiatric Epidemiology*, *48*(2), 295–302. https://doi.org/10.1007/s00127-012-0542-5

Tang, C., Ji, Y., Li, Q., Yao, Z., Cheng, J., He, Y., Liu, X., Pan, R., Wei, Q., Yi, W., & Su, H. (2021). Effects of different heat exposure patterns (accumulated and transient) and schizophrenia hospitalizations: A time-series analysis on hourly temperature basis. *Environmental Science and Pollution Research*, *28*(48), 69160–69170. https://doi.org/10.1007/s11356-021-15371-7

Thawonmas, R., Kim, Y., & Hashizume, M. (2024). Short-term exposure to ambient temperature and the mortality burden of suicide in Japan. *Environmental Research Communications*, *6*(6), 065012. https://doi.org/10.1088/2515-7620/ad4c3f

Thawonmas, R., Kim, Y., & Hashizume, M. (2025). Ambient Temperature and Suicide Risk in Thailand: Evidence from Chiang Mai and Bangkok Provinces. *Environment & Health*, *3*(5), 560–570. https://doi.org/10.1021/envhealth.4c00153

Ulrich, S. E., Sugg, M. M., Guignet, D., & Runkle, J. D. (2025). Mental health disparities among maternal populations following heatwave exposure in North Carolina (2011–2019): A matched analysis. *The Lancet Regional Health - Americas*, *42*, 100998. https://doi.org/10.1016/j.lana.2025.100998

Wang, S., Li, T., & Rajagopalan, P. (2025). Impact of heat exposure on health outcomes among older adults in India: An analysis across ten states. *International Journal of Environmental Health Research*, 1–13. https://doi.org/10.1080/09603123.2025.2461115

Wang, S., Zhang, X., Xie, M., Zhao, D., Zhang, H., Zhang, Y., Cheng, Q., Bai, L., & Su, H. (2018). Effect of increasing temperature on daily hospital admissions for schizophrenia in Hefei, China: A time-series analysis. *Public Health*, *159*, 70–77. https://doi.org/10.1016/j.puhe.2018.01.032

Wang, W., Hao, Y., Peng, M., Yan, J., Xu, L., Yu, H., Yang, Z., & Meng, F. (2025). Individual and combined effects of heatwaves, air pollution, green spaces, and blue spaces on depressive symptoms incidence. *Journal of Environmental Psychology*, *105*, 102684. https://doi.org/10.1016/j.jenvp.2025.102684

Wells, G., Shea, B., O’Connell, D., Peterson, J., Welch, V., Losos, M., & Tugwell, P. (2012). *The Newcastle-Ottawa Scale (NOS) for assessing the quality if nonrandomized studies in meta-analyses*. https://doi.org/10.1207/s15551407vcq1302_4

Xu, R., Zhao, Q., Coelho, M. S. Z. S., Saldiva, P. H. N., Abramson, M. J., Li, S., & Guo, Y. (2020). Socioeconomic level and associations between heat exposure and all-cause and cause-specific hospitalization in 1,814 Brazilian cities: A nationwide case-crossover study. *PLOS Medicine*, *17*(10), e1003369. https://doi.org/10.1371/journal.pmed.1003369

Yoo, E., Eum, Y., Gao, Q., & Chen, K. (2021). Effect of extreme temperatures on daily emergency room visits for mental disorders. *Environmental Science and Pollution Research*, *28*(29), 39243–39256. https://doi.org/10.1007/s11356-021-12887-w

Yoo, E., Eum, Y., Roberts, J. E., Gao, Q., & Chen, K. (2021). Association between extreme temperatures and emergency room visits related to mental disorders: A multi-region time-series study in New York, USA. *Science of The Total Environment*, *792*, 148246. https://doi.org/10.1016/j.scitotenv.2021.148246

Zhang, H., Li, X., Wang, S., Wu, T., Yang, X., Wang, N., Huang, L., Feng, Z., He, Z., Wang, Q., Ling, L., & Zhou, W. (2024). Association Between Extreme Heat and Outpatient Visits for Mental Disorders: A Time‐Series Analysis in Guangzhou, China. *GeoHealth*, *8*(10). https://doi.org/10.1029/2024gh001165

Zhao, R., Chen, S., Wang, W., Huang, J., Wang, K., Liu, L., & Wei, S. (2017). The impact of short-term exposure to air pollutants on the onset of out-of-hospital cardiac arrest: A systematic review and meta-analysis. *International Journal of Cardiology*, *226*, 110–117. https://doi.org/10.1016/j.ijcard.2016.10.053

Zhong, Z., Xu, J., Liu, Z., Tang, X., Zhang, J., Xie, T., Liu, Y., Huang, C., Zhu, D., & Wang, J. (2025). The impact of different types of extreme temperature events on mental disorders: A case-crossover study in Anhui Province, China. *Environmental Research*, *277*, 121526. https://doi.org/10.1016/j.envres.2025.121526

Zhou, Q., Huang, X., Su, L., Tang, X., Qin, Y., Huo, Y., Zhou, C., Lan, J., Zhao, Y., Huang, Z., Huang, G., & Wei, Y. (2024). Immediate and delayed effects of environmental temperature on schizophrenia admissions in Liuzhou, China, 2013–2020: A time series analysis. *International Journal of Biometeorology*, *68*(5), 843–854. https://doi.org/10.1007/s00484-024-02629-1

Zhou, Y., Ji, A., Tang, E., Liu, J., Yao, C., Liu, X., Xu, C., Xiao, H., Hu, Y., Jiang, Y., Li, D., Du, N., Li, Y., Zhou, L., & Cai, T. (2023). The role of extreme high humidex in depression in Chongqing, China: A time series-analysis. *Environmental Research*, *222*, 115400. https://doi.org/10.1016/j.envres.2023.115400
